# Presynaptically Localized Cyclic GMP-Dependent Protein Kinase 1 Is a Key Determinant of Spinal Synaptic Potentiation and Pain Hypersensitivity

**DOI:** 10.1371/journal.pbio.1001283

**Published:** 2012-03-13

**Authors:** Ceng Luo, Vijayan Gangadharan, Kiran Kumar Bali, Rou-Gang Xie, Nitin Agarwal, Martina Kurejova, Anke Tappe-Theodor, Irmgard Tegeder, Susanne Feil, Gary Lewin, Erika Polgar, Andrew J. Todd, Jens Schlossmann, Franz Hofmann, Da-Lu Liu, San-Jue Hu, Robert Feil, Thomas Kuner, Rohini Kuner

**Affiliations:** 1 Pharmacology Institute, University of Heidelberg, Heidelberg, Germany; 2 Institute of Neuroscience, Fourth Military Medical University, Xi'an, China; 3 Molecular Medicine Partnership Unit of the European Molecular Biology Laboratory and the University of Heidelberg Medical Faculty, Heidelberg, Germany; 4 Pharmazentrum Frankfurt, Klinikum der Johann Wolfgang Goethe Universität, Frankfurt am Main, Germany; 5 Interfakultäres Institut für Biochemie, Universität Tübingen, Tübingen, Germany; 6 Max Delbrück Center for Molecular Medicine, Berlin-Buch, Germany; 7 Spinal Cord Group, West Medical Building, University of Glasgow, Glasgow, United Kingdom; 8 FOR 923, Institut für Pharmakologie und Toxikologie, Technische Universität München, Munich, Germany; 9 Institute for Anatomy and Cell Biology, University of Heidelberg, Heidelberg, Germany; Mount Sinai School of Medicine, United States of America

## Abstract

Synaptic long-term potentiation (LTP) at spinal neurons directly communicating pain-specific inputs from the periphery to the brain has been proposed to serve as a trigger for pain hypersensitivity in pathological states. Previous studies have functionally implicated the NMDA receptor-NO pathway and the downstream second messenger, cGMP, in these processes. Because cGMP can broadly influence diverse ion-channels, kinases, and phosphodiesterases, pre- as well as post-synaptically, the precise identity of cGMP targets mediating spinal LTP, their mechanisms of action, and their locus in the spinal circuitry are still unclear. Here, we found that Protein Kinase G1 (PKG-I) localized presynaptically in nociceptor terminals plays an essential role in the expression of spinal LTP. Using the Cre-lox P system, we generated nociceptor-specific knockout mice lacking PKG-I specifically in presynaptic terminals of nociceptors in the spinal cord, but not in post-synaptic neurons or elsewhere (SNS-PKG-I^−/−^ mice). Patch clamp recordings showed that activity-induced LTP at identified synapses between nociceptors and spinal neurons projecting to the periaqueductal grey (PAG) was completely abolished in SNS-PKG-I^−/−^ mice, although basal synaptic transmission was not affected. Analyses of synaptic failure rates and paired-pulse ratios indicated a role for presynaptic PKG-I in regulating the probability of neurotransmitter release. Inositol 1,4,5-triphosphate receptor 1 and myosin light chain kinase were recruited as key phosphorylation targets of presynaptic PKG-I in nociceptive neurons. Finally, behavioural analyses in vivo showed marked defects in SNS-PKG-I^−/−^ mice in several models of activity-induced nociceptive hypersensitivity, and pharmacological studies identified a clear contribution of PKG-I expressed in spinal terminals of nociceptors. Our results thus indicate that presynaptic mechanisms involving an increase in release probability from nociceptors are operational in the expression of synaptic LTP on spinal-PAG projection neurons and that PKG-I localized in presynaptic nociceptor terminals plays an essential role in this process to regulate pain sensitivity.

## Abstract 2

Electrophysiological and behavioral experiments in mice reveal that a cGMP-dependent kinase amplifies neurotransmitter release from peripheral pain sensors, potentiates spinal synapses, and leads to exaggerated pain.

## Author Summary

Pain is an important physiological function that protects our body from harm. Pain-sensing neurons, called nociceptors, transduce harmful stimuli into electrical signals and transmit this information to the brain via the spinal cord. When nociceptors are persistently activated, such as after injury, the connections they make with neurons in the spinal cord are altered in a process called synaptic long-term potentiation (LTP). In this study, we examine the molecular and cellular mechanisms of LTP at synapses from nociceptors onto spinal neurons. We use multiple experimental approaches in mice, from genetic to behavioural, to show that this form of LTP involves presynaptic events that unfold in nociceptors when they are repetitively activated. In particular, an enzyme activated by the second messenger cGMP, referred to as Protein Kinase G-I, phosphorylates presynaptic proteins and increases the release of neurotransmitters from nociceptor endings in the spinal cord. When we genetically silence Protein Kinase G-I or block its activation in nociceptors, inflammatory pain is markedly reduced at the behavioural level. These results clarify basic mechanisms of pathological pain and pave the way for new therapeutic approaches.

## Introduction

Plasticity in peripheral nociceptors and their synapses with spinal neurons has been proposed as a cellular basis for the development and maintenance of pain hypersensitivity following peripheral inflammation or nerve injury [1]–[3]. Activation of nociceptive nerve afferents at frequencies relevant to pathological pain states can trigger long-term potentiation (LTP) at spinal synapses between nociceptor terminals and spinal neurons projecting nociceptive information to the brain [4],[5]. Importantly, this form of synaptic plasticity can be evoked by asynchronous activation of nociceptors in vivo [5], occurs in humans [6], and is functionally associated with a sensation of exaggerated pain [5],[6]. Although there is evidence for a requirement of post-synaptic calcium-dependent mechanisms in the induction of LTP at this synapse [5], the precise mechanisms underlying the expression of spinal LTP are not entirely clear [7].

Synaptic LTP evoked by natural, asynchronous low-rate discharges in C-nociceptors on spino-PAG neurons was recently shown to constitute a very fitting correlate of spinal amplification phenomena underlying inflammatory pain [5],[7]. This form of synaptic change has been reported to involve activation of NMDA receptors, NO release, and synthesis of cGMP [5],[7]. However, which of the diverse targets of cGMP come into play at this synapse and how they mechanistically bring about long-lasting changes in the transfer of nociceptive information between the nociceptors and spinal neurons projecting to the brain is not understood so far. Furthermore, very little is known about exactly how neural circuits involved in pain processing are modulated by cGMP and which cellular and molecular processes underlie these changes.

Studies on several different biological systems have shown that cGMP regulates multiple cellular targets, including diverse cGMP-gated ion channels, such as cyclic nucleotide-gated (CNG) and hyperpolarization-activated cyclic nucleotide-gated (HCN) channels, the cGMP-dependent protein kinases, PKG-I/cGK-I and PKG-II/cGK-II, as well as diverse phosphodiesterases (PDEs) [8],[9]. Nearly all of these molecular targets of cGMP are expressed in nociceptive pathways and may potentially contribute to the key role of cGMP in synaptic potentiation in the spinal cord. Amongst these targets, PKG-I has emerged as a key mediator of cGMP functions in smooth muscle and platelet function [8]. The α-isoform of PKG-I has been reported to be expressed very highly in the primary sensory neurons in the dorsal root ganglia (DRG) over developmental [10] and adult stages [11], and several regions in the brain and the spinal cord also express PKG-I [12],[13]. Pharmacological and genetic studies in global, constitutive mutant mice have linked PKG-I to the development of the nociceptive circuitry as well as to spinal mechanisms of hyperalgesia [14].

Based upon this background, this study was designed with two goals in mind. First, it addressed the potential involvement of presynaptic mechanisms in the expression of synaptic potentiation on spinal projection neurons, which has not been explored or described previously. Second, it aimed to explore a potential role for PKG-I localized presynaptically in the spinal terminals of nociceptors in spinal potentiation and to clarify cellular and molecular mechanisms underlying these processes. We reasoned that the use of a conditional, region-specific gene deletion strategy to specifically manipulate presynaptic mechanisms might constitute an unambiguous approach towards addressing the above questions. Our results show that spinal synaptic potentiation triggered by nociceptor activation is associated with a long-lasting change in the probability of neurotransmitter release from spinal terminals of nociceptors. Using viable, developmentally normal transgenic mice lacking the PKG-I specifically in nociceptors with preserved expression in spinal neurons, brain, and all other organs, we demonstrate here that PKG-I localised in nociceptor terminals constitutes a key mediator of synaptic LTP and that its activation is functionally associated with pain hypersensitivity in vivo.

## Results

### Mice Lacking PKG-I Conditionally in a Nociceptor-Specific Manner

**Figure 1:**
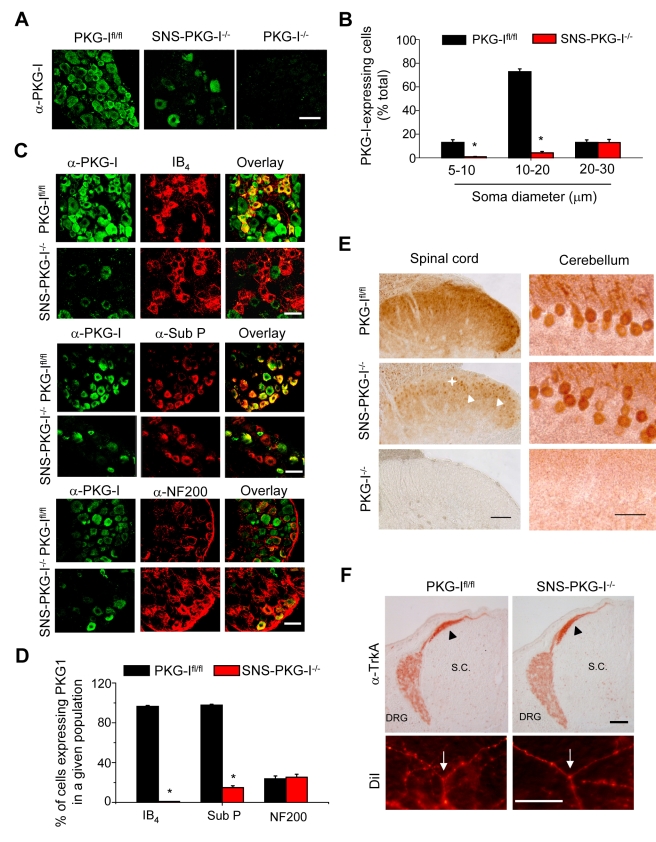
Nociceptor-specific deletion of PKG-I and lack of developmental defects in SNS-PKG-I^−/−^ mice. (A) Immunoreactivity for an anti-PKG-I antibody was detected widely in DRG neurons of mice carrying floxed PKG-I alleles (PKG-I^fl/fl^) and was lost either selectively in SNS-PKG-I^−/−^ mice or entirely in global PKG-I^−/−^ mice. (B) Quantitative size analysis of neurons of the dorsal root ganglia (DRG) expressing PKG-I in SNS-PKG-I^−/−^ mice and PKG-I^fl/fl^ littermates showing specific loss of PKG-I in small-diameter neurons in SNS-PKG-I^−/−^ mice. Data in panels represent mean ± S.E.M.; *n* = 10–15 DRG sections each. (C, D) Typical examples (C) and quantitative summary (D) from dual immunofluorescence experiments showing that in SNS-PKG-I^−/−^ mice, PKG-I immunoreactivity is abrogated from nociceptors, i.e. Isolectin-B_4_-positive (IB_4_) or Substance P-positive (α-Sub P) neurons, but retained in neurofilament 200-positive (α-NF200) large diameter neurons, in comparison with control littermates (PKG-I^fl/fl^). *n* = 10–15 DRG sections each; * *p*<0.001, ANOVA, post hoc Fisher's test. (E) Typical examples of anti-PKG-I immunostaining in the brain (cerebellar Purkinje neurons are shown) and spinal dorsal horns of SNS-PKG-I^−/−^ mice, their PKG-I^fl/fl^ littermates, and global PKG-I^−/−^ mice. Spinal dorsal horns of SNS-PKG-I^−/−^ mice showed a loss of anti-PKG-I immunoreactivity in the superficial neuropil (+), but distinct preservation of signals in cell bodies (arrowheads). (F) Typical examples of patterning of Trk-A-expressing sensory afferents (arrowheads in upper panels) and T-branching of DiI-labelled sensory afferents (arrows in lower panels) in the spinal cord in PKG-I^fl/fl^ mice and SNS-PKG-I^−/−^ mice at embryonic day 13.5 (E13.5). Scale bars represent 50 µm in panel A, 50 µm in panels A and C, 100 µm for spinal cord and 50 µm for cerebellum in panel E, and 100 µm in panel F. DRG, dorsal root ganglia; S.C., spinal cord.

Mice lacking PKG-I specifically in a primary nociceptor-specific manner (SNS-PKG-I^−/−^) were generated via Cre/loxP-mediated recombination by mating mice carrying the floxed *prkg1* allele (PKG-I^fl/fl^) [15] with a mouse line expressing Cre recombinase under control of the Na_v_1.8 promoter (SNS-Cre) [16]. We have previously demonstrated that SNS-Cre mice enable gene recombination commencing at birth selectively in nociceptive (Na_v_1.8-expressing) sensory neurons, without affecting gene expression in the spinal cord, brain, or any other organs in the body [16],[17]. An anti-PKG-I antibody [18] yielded specific staining in wild-type dorsal root ganglia (DRG), but not in those from global PKG-I^−/−^ mice [19], thereby revealing Cre/loxP-mediated deletion of PKG-I in DRG of SNS-PKG-I^−/−^ mice (Figure 1A). Quantitative size-frequency analysis revealed that a majority of DRG neurons expressing PKG-I in wild-type mice are small-diameter neurons, which show a near complete loss of PKG-I expression in SNS-PKG-I^−/−^ mice (Figure 1B; *p*<0.001). In contrast, a few large-diameter neurons showed low levels of anti-PKG-I immunoreactivity in DRGs of PKG-I^fl/fl^, which was entirely retained in SNS-PKG-I^−/−^ mice (Figure 1A and B). Confocal analysis of dual immunofluorescence experiments revealed PKG-I immunoreactivity in nearly all Isolectin-B_4_ (IB_4_)-labelled non-peptidergic nociceptors and substance P-expressing peptidergic nociceptors in PKG-I^fl/fl^ mice, both of which are selectively lost in SNS-PKG-I^−/−^ mice (typical examples in Figure 1C and quantitative summary in Figure 1D). In contrast, large-diameter neurofilament-200-immunoreactive neurons entirely retained PKG-I expression in the SNS-PKG-I^−/−^ mice (Figure 1C,D). Taken together, these results show that PKG-I is normally expressed in nearly all nociceptors and is selectively lost from these neurons, but not from tactile-responsive and proprioceptive DRG neurons, in SNS-PKG-I^−/−^ mice.

We found that PKG-I expression is entirely unaltered in the brains of SNS-PKG-I^−/−^ mice (an example of expression in cerebellar purkinje neurons is shown in Figure 1E, right panel), whereas global PKG-I^−/−^ mice demonstrated a complete loss of anti-PKG-I immunoreactivity (Figure 1E, right panel). In the spinal cord of SNS-PKG-I^−/−^ mice, anti-PKG-I immunoreactivity was decreased selectively in the superficial dorsal laminae, which represent termination zones of the nociceptive afferents, as would be expected from SNS-Cre-mediated gene deletion in nociceptors (Figure 1E, left panel). In contrast, neurons in the spinal cord entirely maintained immunoreactivity for PKG-I and appeared particularly conspicuous (arrowheads in Figure 1E, left panel) due to the loss of PKG-I labelling in afferent terminals in SNS-PKG-I^−/−^ mice. Furthermore, anti-Cre immunohistochemistry as well as Western blot analysis with anti-PKG-I antibody confirmed that SNS-PKG-I^−/−^ mice show a DRG-specific loss of PKG-I while retaining expression in the spinal cord and brain (Figure S1).

In contrast to global PKG-I^−/−^ mice, which typically demonstrate lethality in the first few weeks of life, SNS-PKG-I^−/−^ mice were normal, fertile, and showed a normal life expectancy. In contrast to defects reported in global PKG-I^−/−^ mice [10], SNS-PKG-I^−/−^ mice showed normal early targeting of TrkA-expressing primary afferents arising from the DRG (arrowheads in Figure 1F, upper panels) in the developing spinal dorsal horn at embryonic day 13 (E13). Unlike global PKG-I^−/−^ mice [10], SNS-PKG-I^−/−^ mice did not show defects in T-branching of DiI-labelled primary afferents in the spinal cord over embryonic developmental stages (arrows in Figure 1F, lower panels). Similarly, central and peripheral patterning of peptidergic or non-peptidergic nociceptors was normal in adult SNS-PKG-I^−/−^mice, as revealed by immunostaining for substance P and binding to IB_4_, respectively, in the spinal dorsal horns and skin (Figure S2A). Because peptidergic mechanisms have been suggested to play an important role in spinal LTP [5], we ascertained that SNS-PKG-I^−/−^ mice are not different from control mice with respect to the abundance of substance P in the spinal circuitry. Control and knockout mice exhibited the same prevalence of substance P-immunoreactive cells within DRG (33%±5% versus 29%±3%, respectively), which were not significantly different from each other (*p*>0.05, Student's *t* test). Moreover, the level of substance P immunoreactivity was similar in the superficial spinal dorsal horn across genotypes (mean intensities in PKG-I^fl/fl^ mice and SNS-PKG-I^−/−^ mice were 50±3 and 51±3 arbitrary units, respectively). Importantly, confocal microscopy revealed normal density of synapses between substance P-containing nociceptive afferents and PSD-95-positive puncta (representing postsynaptic aspects of glutamatergic synapses) in the spinal dorsal horns of SNS-PKG-I^−/−^ mice as compared to PKG-I^fl/fl^ mice (examples and quantification in Figure S2B). Finally, we addressed the internalization of NK1 receptors on spinal lamina I neurons following peripheral nociceptive stimulation in vivo, which has been demonstrated to be a clear indicator of nociceptive activity-induced synaptic release of substance P [20]. As shown in Figure S2C, application of a 52°C heat stimulus for 20 s to the plantar paw surface led to internalization of NK1 receptors in lamina I neurons of L3/L4 segments to a similar extent in SNS-PKG-I^−/−^ and PKG-I^fl/fl^ mice (quantification in Figure S2D). Unlike global PKG-I^−/−^ mice [14], SNS-PKG-I^−/−^ mice showed a normal lamination of the spinal cord over early postnatal stages (Figure S2E). Thus, the multiple developmental defects in the patterning of sensory afferents and spinal lamination that have been reported in global PKG-I^−/−^ mice were not observed in SNS-PKG-I^−/−^ mice.

### Loss of PKG-I in Presynaptic C-Fiber Terminals, But Not Postsynaptically in Spino-PAG Neurons, Precludes Expression of Spinal LTP without Altering Basal Transmission or Excitability

**Figure 2:**
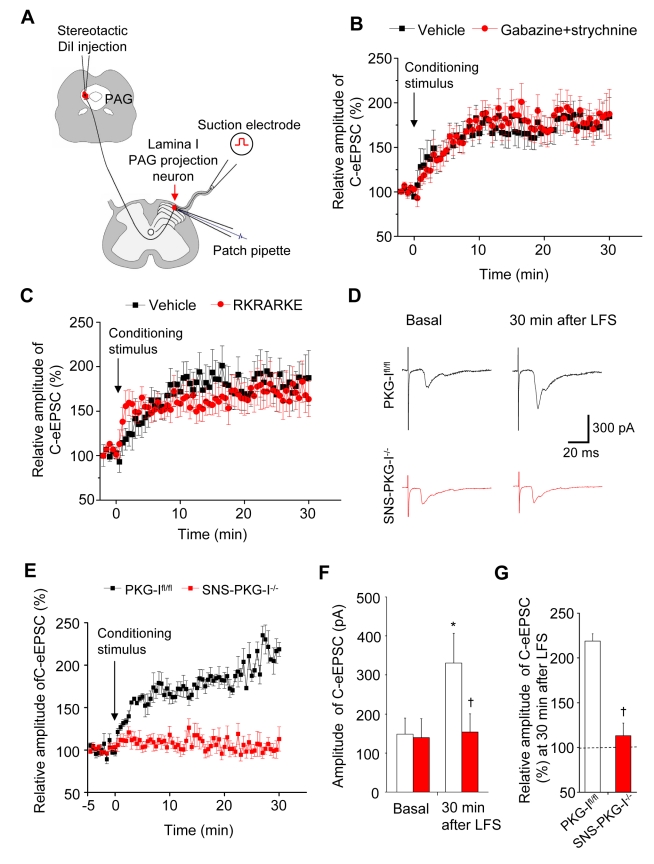
Contribution of PKG-I to synaptic long-term potentiation (LTP) at contacts between C-nociceptors and spinal-PAG projection neurons. (A) Schematic representation of the experimental approach for dorsal root stimulation and whole-cell patch clamp recordings from spino-PAG projection neurons in lamina I. (B, C) Synaptic LTP was observed following conditioning low-frequency stimulation (2 Hz) of dorsal roots in wild-type mice. LTP was preserved upon spinal blockade of inhibitory neurotransmission (panel B) or upon blockade of PKG-I specifically in the postsynaptic neuron via application of a non-permeant inhibitor in the patch pipette (panel C); *n* = 11–19 slices each. (D–G) Representative traces (D), time course (E), and quantitative summary of LTP (F, G) evoked by conditioning low-frequency stimulation (2 Hz) of dorsal roots in PKG-I^fl/fl^ mice, but not in SNS-PKG-I^−/−^ mice; *n* = 12 slices per genotype. All data are represented as mean ± S.E.M. * *p*<0.05 as compared to basal and † *p*<0.05 as compared to PKG-I^fl/fl^ mice, ANOVA followed by post hoc Fisher's test.

To address activity-dependent plasticity at spinal synapses, we recorded C-fiber-evoked synaptic LTP on spinal lamina I neurons projecting to the periaqueductal grey (PAG), which were retrogradely labelled upon stereotactic injection of DiI in the PAG (the experimental scheme is shown in Figure 2A and an example of a labelled cell is shown in Figure S3A) [5]. In spinal-PAG projection neurons of wild-type mice, a conditioning low frequency stimulation of 2 Hz for 2 min produced synaptic LTP of monosynaptic C-fiber evoked EPSCs by more than 200% at 30 min (Figure 2B). LTP at these synapses was preserved in the presence of strychnine and gabazine, which block glycinergic and GABAergic inhibitory neurotransmission, respectively (Figure 2B). Similar results were obtained upon using another standard blocker of GABAergic neurotransmission, bicuculline, in combination with strychnine (Figure S3B). Hence, LTP does not manifest due to primary afferent depolarization mediated by presynaptic GABA receptors or disinhibition of the postsynaptic neuron. To test whether LTP requires a postsynaptic function of PKG-I, we dialyzed standard PKG-I inhibitors, such as the non-permeant peptide inhibitor RKRARKE [21],[22] or KT5823 [23], into spinal neurons via the patch pipette. These manipulations did not affect the magnitude or duration of C-fiber-evoked LTP at spino-PAG synapses (Figure 2C and Figure S3C), suggesting that PKG-I localized postsynaptically in spino-PAG projection neurons does not play a role in LTP at this synapse.

To assess the role of PKG-I localized presynaptically in spinal nociceptor terminals, we then analysed PKG-I^fl/fl^ mice and SNS-PKG-I^−/−^ mice. In spinal-PAG projection neurons of PKG-I^fl/fl^ mice, a conditioning low frequency stimulation of 2 Hz for 2 min produced LTP with a magnitude of more than 200% at 30 min and more than 300% by 60 min (typical examples of time course and EPSC traces are given in Figure 2D,E). Prior to the conditioning stimulus, baseline values of C-fiber-evoked EPSCs stayed constant over the period of recording in both genotypes (Figure 2D,E). In striking contrast to PKG-I^fl/fl^ mice, the conditioning stimulus did not evoke LTP in spinal-PAG projection neurons in SNS-PKG-I^−/−^ mice (Figure 2D,E; see Figure 2F and 2G for quantitative summary at 30 min post-conditioning stimulus; *p*<0.001; at least 13 neurons from each genotype were tested).

**Figure 3:**
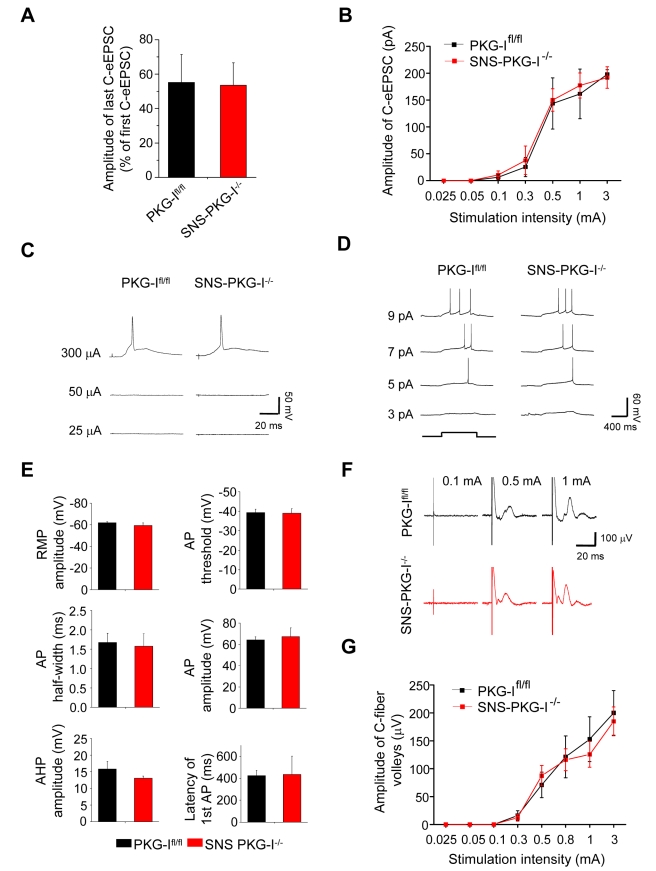
Analysis of basal neurotransmission and properties of spinal-PAG projection neurons in spinal slices derived from SNS-PKG-I^−/−^ mice and their PKG-I^fl/fl^ littermates. (A, B) Short-term depression during the conditioning train of low-frequency stimulation (A) and input-output curves for basal synaptic transmission between C-fibers and spinal-PAG projections neurons (B) were not different between PKG-I^fl/fl^ mice and SNS-PKG-I^−/−^ mice (*n* = 12 each). (C, D) Activation properties of spinal neurons are not altered in SNS-PKG-I^−/−^ mice as compared to their PKG-I^fl/fl^ littermates. Dorsal root stimulation threshold for evoking action potentials (C) in spinal-PAG projection neurons and spiking properties of spinal-PAG projection neurons upon direct current injection (D) are similar in SNS-PKG-I^−/−^ mice and their PKG-I^fl/fl^ littermates. (E) Resting membrane potential (RMP), action potential (AP) properties, such as threshold, amplitude, half-width, and the latency of first AP, and amplitude of after hyperpolarization (AHP) were similar between genotypes (*p*>0.05; Student's *t* test). (F, G) Typical traces (F) and magnitude of C-fiber volleys in L4/L5 roots recorded at different intensities of stimulation are comparable in SNS-PKG-I^−/−^ mice and their PKG-I^fl/fl^ littermates (*n* = 16 each).

For a clear interpretation of these data, it is imperative to address how basal nociceptive transmission at these synapses is affected in SNS-PKG-I^−/−^ mice. Analysis of EPSC magnitude evoked by the first and the last pulse of the conditioning train revealed short-term depression of evoked EPSCs during the conditioning train, which was equivalent in PKG-I^fl/fl^ mice and SNS-PKG-I^−/−^ mice (Figure 3A; *p* = 0.95), showing that the conditioning stimulus was equally effective in mice from both groups. Furthermore, basal C-fiber-evoked EPSCs were comparable between PKG-I^fl/fl^ and SNS-PKG-I^−/−^ mice. We also established detailed input-output curves representing the relationship between the intensity of dorsal root stimulation and evoked EPSCs in the absence of a conditioning stimulus and found no differences between PKG-I^fl/fl^ and SNS-PKG-I^−/−^ mice (Figure 3B; *p* = 0.74). Furthermore, the intensity of dorsal root stimulation required to elicit an action potential in post-synaptic spinal-PAG projection neurons was identical in PKG-I^fl/fl^ and SNS-PKG-I^−/−^ mice (an example is shown in Figure 3C). The intact nature of responsiveness in spinal-PAG projection neurons was demonstrated by similarities in their activation profiles upon current injection in SNS-PKG-I^−/−^ mice and their PKG-I^fl/fl^ littermates (an example is shown in Figure 3D). In all of the above experiments, quantitative analyses revealed that resting membrane potential, action potential width, delay after stimulation artefact, action potential threshold, delay for generation of first action potential (latency to first AP), as well as amplitude of after hyperpolarisation (AHP) were similar in SNS-PKG-I^−/−^ mice and PKG-I^fl/fl^ mice (Figure 3E; *p*>0.05 in all cases, Student's *t* test). Finally, as an additional indicator of the number of fibers activated during electrical stimulation, we recorded fiber volleys in input/output measurements. Recording C-fiber volleys in the L4 and L5 dorsal roots derived from PKG-I^fl/fl^ and SNS-PKG-I^−/−^ mice revealed typical responses, which increased in amplitude with increasing stimulus intensity (representative traces are shown in Figure 3F). The amplitudes of C-fiber volley responses were not significantly different between PKG-I^fl/fl^ and SNS-PKG-I^−/−^ mice (stimulus-response curves are shown in Figure 3G; *p*>0.05; *n* = 16 per genotype), demonstrating directly that the number of fibers activated upon electrical stimulation was comparable between genotypes and could therefore not explain the failure to evoke synaptic potentiation in SNS-PKG-I^−/−^ mice. These comprehensive analyses show that a presynaptic loss of PKG-I was specifically linked to a failure of activity-dependent potentiation of transmission at synapses between nociceptors and spinal-PAG projection neurons, but not to modulation of basal synaptic transmission.

### Evidence for a Presynaptic Mechanism for Synaptic Potentiation on Spinal-PAG Projection Neurons and a Role for Presynaptic PKG-I

**Figure 4:**
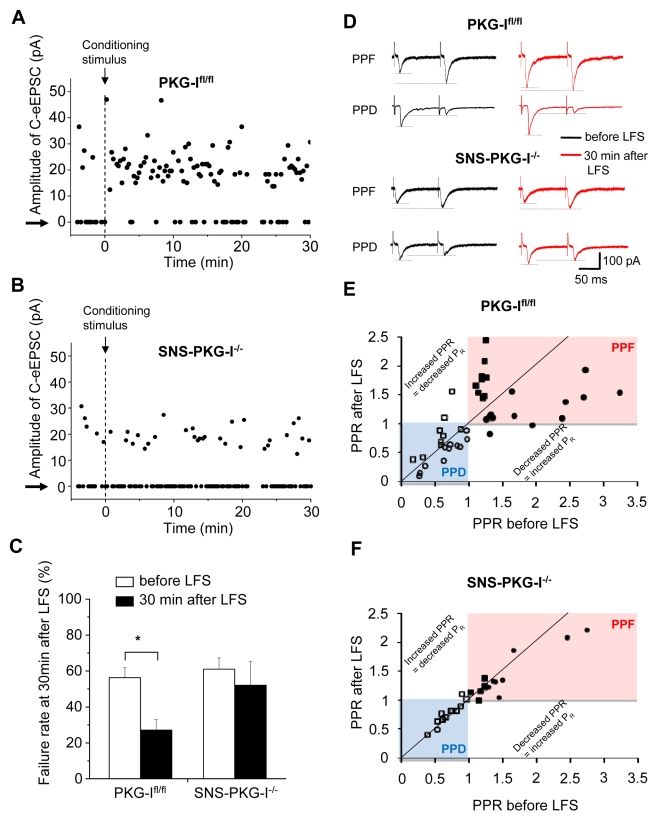
Analysis of a presynaptic component to C-fiber-evoked LTP on spinal-PAG projection neurons. (A, B) Activation of C-nociceptors potentiates synaptic transmission by decreasing the rate of synaptic failures via activation of presynaptic PKG-I. Shown are typical examples of the frequency of synaptic failures (arrows) and synaptic successes (C-fiber-evoked EPSCs) evoked by minimal stimulation of dorsal roots prior to and after application of the conditioning stimulus in PKG-I^fl/fl^ mice (A) and SNS-PKG-I^−/−^ mice (B). (C) Summary of average failure rates upon minimal stimulation of dorsal roots prior to and 30 min following the conditioning stimulus (*n* = 5 slices for PKG-I^fl/fl^ mice and 7 slices for SNS-PKG-I^−/−^ mice). (D–F) Analysis of paired-pulse facilitation (PPF) and paired-pulse depression (PPD) of C-fiber-evoked EPSCs induced by pairs of stimuli at inter-stimulus interval of 110 ms in spino-PAG projection neurons from PKG-I^fl/fl^ and SNS-PKG-I^−/−^ mice. Traces of typical recordings showing PPF or PPD prior to (basal) and at 30 min following low-frequency conditioning stimulation of C-fibers (LFS) are shown in panel D. Paired-pulse ratios (PPR) prior to and at 30 min after conditioning stimulation are plotted in panel E (PKG-I^fl/fl^ mice) and panel F (SNS-PKG-I^−/−^ mice). Values of PPR above and below 1 prior to conditioning stimulation are represented as PPF (pink field) and PPD (blue field), respectively. Note that in PKG-I^fl/fl^ mice, neurons showing low initial PPF show an increase in PPF after conditioning stimulation, whereas neurons showing an initial high PPF show a decrease in PPF following conditioning stimulation, which corresponds to an increase in probability of release. These changes are reduced or do not come about in SNS-PKG-I^−/−^ mice. All data are represented as mean ± S.E.M. * *p*<0.05, ANOVA of random measures followed by post hoc Fisher's test.

Following our observation that a specific presynaptic alteration in PKG-I expression perturbed spinal LTP, we then addressed potential contributions of presynaptic mechanisms at synapses between nociceptors and spinal projection neurons [7],[24]. By recording miniature EPSCs in spinal-PAG projection neurons of control mice, we observed that quantal content varies largely, which is expected since these spinal projection neurons receive multisynaptic inputs from primary afferents as well as spinal interneurons. Furthermore, inputs arising from spinal interneurons are not expected to change in SNS-PKG-I^−/−^ mice since the molecular perturbation is specific to nociceptor terminals. Thus, the net contribution of C-fibers to the population of mEPSCs is difficult to assess because it is unclear which fraction of mEPSCs can be attributed to C-fibers, which then makes detection of potentially small presynaptic changes highly unlikely when performing mini-analysis. To study synaptic events which could be clearly assigned to activation of presynaptic primary afferent fibers alone, we employed a protocol of minimal stimulation, setting the dorsal root stimulation parameters such that a synaptic failure rate of approximately 60% was achieved in recording solution containing 1 mM Ca^2+^ and 5 mM Mg^2+^. The failure rate remained constant over a period of at least 30 min upon repetitive test stimulation in the absence of a conditioning stimulus (55.9%±3.9% pre- and 57.1%±8.1% at 30 min). However, upon application of the conditioning stimulus, minimal stimulation using the same parameters evoked a decrease in the frequency of synaptic failures within a few minutes in slices derived from PKG-I^fl/fl^ mice, indicating a change in the probability of neurotransmitter release; decrease in synaptic failures was accompanied by a corresponding rise in the magnitude of C-fiber-evoked EPSCs (see Figure 4A for typical example and Figure 4C for quantitative summary of C-fiber-evoked EPSCs recorded every 15 s; *n* = 5; *p* = 0.04). In contrast, the rate of synaptic failures did not change significantly following conditioning stimulus in slices derived from SNS-PKG-I^−/−^ mice (see Figure 4B and 4C; *n* = 7; *p* = 0.68). EPSC values at the end of the recording were not significantly elevated as compared to basal values in SNS-PKG-I^−/−^ mice (25±1.7 pA before and 21.1±1.2 pA at 30 min after the conditioning stimulus; *p* = 0.14).

In analogy to studies on hippocampal circuits, although the above evidence for a decrease in failure rate is indicative of increased presynaptic release probability, it has also been linked to unsilencing of silent synapses [25]. Therefore, to further consolidate presynaptic mechanisms, we focussed on the analysis of paired-pulse facilitation (PPF), which represents a short-lasting increase in the second evoked EPSP when it follows shortly after the first and is well accepted as an indication of presynaptic mechanisms of long-term potentiation in the hippocampus [26]. In hippocampal CA1 neurons, PPF can increase as well as decrease in conjunction with LTP in a manner inversely proportional to the PPF prior to the conditioning stimulus [26]. Indeed, we obtained similar results in recordings at spinal synapses between C-fibers and spinal-PAG projection neurons. In spinal slices derived from mice of both genotypes, we found evidence for PPF as well as paired-pulse depression (PPD) prior to the LTP-inducing conditioning stimulus (typical traces are shown in Figure 4D). Whereas a majority of neurons derived from PKG-I^fl/fl^ mice demonstrated a clear change in PPF or PPD following conditioning stimulus, neurons derived from SNS-PKG-I^−/−^ mice did not (see examples in Figure 4D). We then plotted the paired-pulse ratio (PPR) of the entire cohort of recorded neurons at 30 min after conditioning stimulation as a function of the basal PPR recorded prior to the conditioning stimulus (Figure 4E,F). This analysis revealed that neurons in PKG-I^fl/fl^ mice with larger basal values of PPR prior to the conditioning stimulus (indicated by filled round symbols in Figure 4E) consistently showed a decrease in PPR after the conditioning stimulus (Figure 4D), which is indicative of an increase in the probability of release (P_R_, Figure 4E). This drop in PPF following conditioning stimulation did not come about or was reduced in neurons from SNS-PKG-I^−/−^ mice (filled round symbols in Figure 4F). A smaller cohort of synapses in PKG-I^fl/fl^ mice showed an increase in PPF after conditioning stimulation, but this was restricted to neurons with a low magnitude of PPF prior to the conditioning stimulus (i.e., a PPR of about 1.1–1.2, filled square symbols in Figure 4E) and a low expression of LTP (Figure S4A). Again, this change was not observed in the corresponding cohort of neurons in SNS-PKG-I^−/−^ mice (filled square symbols in Figure 4F, Figure S4B). Conversely, in PKG-I^fl/fl^mice, higher magnitudes of LTP (i.e., between 150% and 350%, indicated by black frame in Figure S4A) were consistently associated with a decrease in PPR, which is indicative of an increase in release probability. Neither LTP nor consistent changes in PPR were observed in SNS-PKG-I^−/−^ mice (Figure S4B). In conclusion, the failure rate analysis and PPR analysis strongly support the inference that the expression of LTP at spino-PAG synapses comes about via presynaptic mechanisms involving an increase in release probability via PKG-I.

### Signalling Targets of PKG-I in Nociceptors, Their Activation upon Nociceptive Stimulation, and Their Role in Spinal Plasticity

In an effort to understand the underlying molecular mechanisms, we then addressed potential substrates for the kinase activity of PKG-I in nociceptors. In particular, we reviewed known substrates of PKG-I in other biological systems and focussed on those for which we hypothesized a role in synaptic transmission. We first set up an assay system for testing involvement of PKG-I substrates in the DRG selectively upon persistent nociceptive stimulation in vivo, using Vasodilator-stimulated phosphoprotein (VASP), a classical target of PKG-I, as an indicator of PKG-I activity [8],[27]. Lysates of L4-L5 DRGs from naïve PKG-I^fl/fl^ and SNS-PKG-I^−/−^ mice showed comparable levels of VASP expression (Figure S5A, basal). Within minutes after persistent nociceptive stimulation via injection of formalin in the hindpaw, L4-L5 DRGs from PKG-I^fl/fl^ mice showed a striking phosphorylation of VASP at Serine 239 (typical examples in Figure S5A and summary in Figure S5B,C; see Text S1 for details; *p* = 0.03 as compared to basal). This was markedly reduced in formalin-injected SNS-PKG-I^−/−^ mice (Figure S5A,B; *p* = 0.18 as compared to basal SNS-PKG-I^−/−^ mice and 0.03 as compared to formalin-injected PKG-I^fl/fl^ mice). These results show that persistent activation of nociceptors leads to rapid signalling via PKG-I in DRG neurons in vivo, which is lost in SNS-PKG-I^−/−^ mice, as expected.

**Figure 5:**
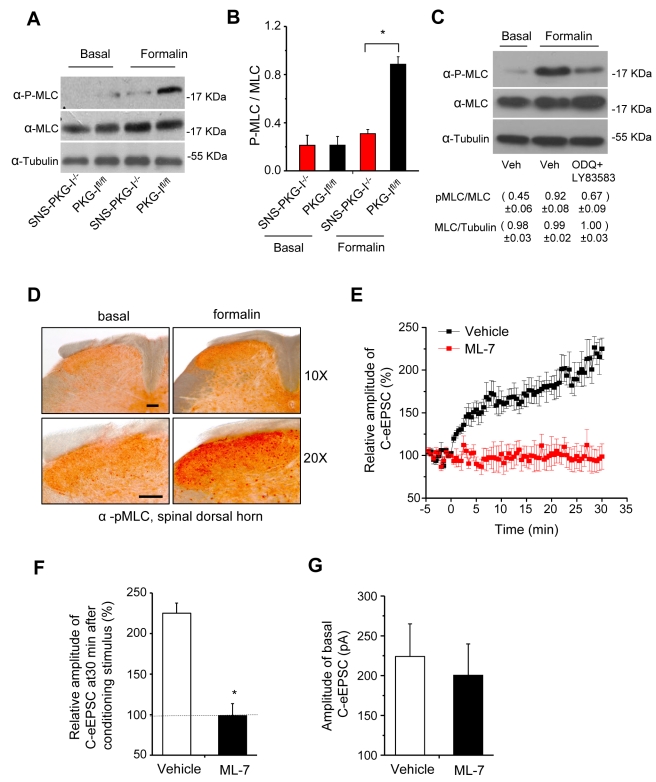
Nociceptive activity-driven, PKG-I-dependent phosphorylation of myosin light chains (MLC) in DRG in vivo and its contribution to synaptic potentiation at contacts between C-nociceptors and spinal-PAG projection neurons. (A, B) A typical example (A) and quantitative summary (B) of levels of phosphorylated MLC in L4-L5 DRGs of SNS-PKG-I^−/−^ mice and PKG-I^fl/fl^ littermates in the naïve state or following formalin injection in the hindpaws. (C) Formalin-induced increase in MLC phosphorylation in L4-L5 DRGs is reduced in wild type mice treated with inhibitors of cGMP synthesis, ODQ (25 mg/kg body weight), and LY83583 (12.5 mg/kg body weight). Mean values from three experiments are depicted below the representative Western blot. (D) Photomicrographic images showing an increase in immunoreactivity for phosphorylated myosin light chains in the spinal dorsal horns in wild-type mice following injection of formalin in the hindpaw. Note increased staining in superficial neuropil as well as cell bodies. Scale bars = 100 µm. (E, F) Time-course (E) and quantitative summary at 30 min (F) after the conditioning stimulus of the blockade of spinal synaptic potentiation by ML-7, an inhibitor of myosin light chain kinase (MLCK) (*n* = 8 slices/group). (G) MLCK blockade does not significantly affect basal transmission upon dorsal root stimulation (3 mA).

Using this assay system, we then addressed another key target of PKG-I, which has been mainly studied so far mechanistically in smooth muscle cells. Dephosphorylation of myosin light chains (MLC) [28] via PKG-I-dependent phosphorylation and activation of myosin light chain phosphatase in smooth muscle cells is a decisive mechanism underlying NO-mediated vasodilation [8]. Following formalin injection in the paw, we observed a strong phosphorylation of MLC in L4-L5 DRGs from PKG-I^fl/fl^ mice, which was found to be lacking in formalin-treated SNS-PKG-I^−/−^ mice (Figure 5A and 5B). These differences did not arise due to differences in expression levels of MLC between SNS-PKG-I^−/−^ mice and PKG-I^fl/fl^ mice (Figure 5B; Figure S5C). This finding was unexpected because it suggests a role for PKG-I in increasing MLC phosphorylation in DRG neurons, which is contrary to the classical role ascribed to PKG-I in MLC dephosphorylation. We reasoned that if our findings hold true, synthesis of cGMP ought to be a critical intermediate step in activity-dependent MLC phosphorylation in DRG neurons. Indeed, in mice pre-treated with an inhibitor of the soluble guanylyl cyclase, ODQ, and a pan-inhibitor of membrane-bound guanylyl cyclases, LY83583, via intrathecal application, formalin-induced MLC phosphorylation in L4-L5 DRGs was strongly reduced (see examples in Figure 5C; quantitative summary from three experiments is given below the Western blot). Immunohistochemistry revealed that nociceptor activation-induced increase in MLC phosphorylation occurred in the spinal termination zone of nociceptors (lamina I and II) as well as in spinal neurons (Figure 5D).

Interestingly, synaptic potentiation induced by a conditioning stimulus on spinal-PAG projection neurons was abolished in the presence of ML-7, an inhibitor of MLCK (Figure 5E and Figure 5F; *p* = 0.004 as compared to vehicle-treated control slices). Furthermore, consistent with our observations in SNS-PKG-I^−/−^ mice, inhibition of MLC phosphorylation did not affect basal transmission at this synapse (Figure 5G; *p* = 0.761). Thus, the PKG-I target, pMLC, is functionally linked to potentiation of synaptic transmission in nociceptive laminae.

**Figure 6:**
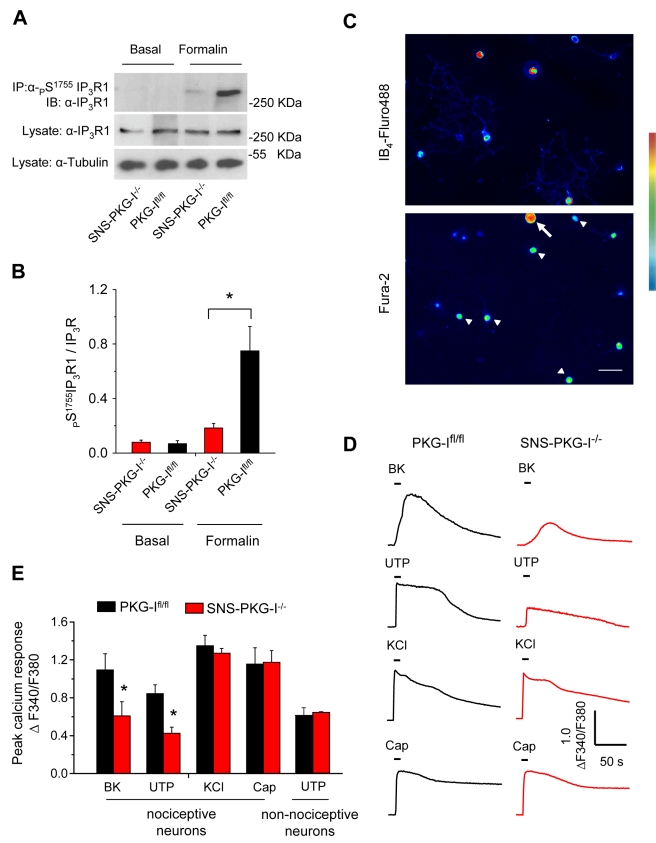
PKG-I mediates nociceptive activity-dependent phosphorylation of Inositol 1,4,5-triphosphate receptor 1 (IP_3_R1) in DRG and potentiates evoked calcium transients. (A, B) Typical examples (A) and quantitative summary (B) of levels of immunoprecipitated IP_3_R1 phosphorylated at serine 1755 or total IP_3_R1 in L4-L5 DRG lysates derived from naïve or formalin-injected PKG-I^fl/fl^ and SNS-PKG-I^−/−^ mice. (C–E) Comparison of evoked calcium transients in DRG neurons in culture derived from SNS-PKG-I^−/−^ mice or their control PKG-I^fl/fl^ littermates. (c) Typical examples of Fura2-loaded dissociated DRG neurons, which were co-stained with Fluor 488-conjugated isolectin B4 (IB4) to identify non-peptidergic nociceptive neurons (arrowheads). (D) Typical examples of traces of calcium transients evoked by bath application of bradykinin (BK, 50 nM), UTP (100 µM), KCl (25 mM), and capsaicin (1 µM) in IB4- and Fura2-double-labelled DRG neurons from PKG-I^fl/fl^ and SNS-PKG-I^−/−^ mice. (E) Ratiometric Fura2-based imaging of calcium released upon bath application of bradykinin (BK, 50 nM), UTP (100 µM), KCl (25 mM), and capsaicin (1 µM) in IB4- and Fura2-double-labelled nociceptive DRG neurons from PKG-I^fl/fl^ and SNS-PKG-I^−/−^ mice (9–10 independent culture experiments). Note that UTP-induced transients in non-nociceptive large-diameter neurons (e.g., arrow in panel C) are not different across genotypes. * *p*<0.05, ANOVA of random measures followed by post hoc Fisher's test. Scale bar represents 20 µm in panel C.

Ikeda et al. [5] have reported that inhibition of IP_3_R activation blocks conditioning stimulus-induced synaptic potentiation at synapses between nociceptors and spinal-PAG projection neurons. This is particularly interesting because IP_3_R1 contains a PKG-I-recognition motif at serine 1755 and has been reported to be phosphorylated by PKG-I in vitro, putatively leading to gain of function [29],[30]. We observed that IP_3_R1 is indeed a target of PKG-I in nociceptors and is functionally associated with modulation of calcium release from intracellular stores. In immunoprecipitation experiments from L4-L5 DRGs, formalin-injected PKG-I^fl/fl^ mice demonstrated highly enhanced serine 1755 phosphorylation of IP_3_R1 over the basal state; this effect was markedly reduced in DRGs obtained from formalin-injected SNS-PKG-I^−/−^ mice (Figure 6A,B), although expression levels of IP_3_R1 were comparable between SNS-PKG-I^−/−^ mice and PKG-I^fl/fl^ mice (Figure S5D).

PKG-I-mediated phosphorylation of serine 1755 in IP_3_R1 has been suggested to positively modulate IP_3_R1 activity in heterologous test systems [30]. We observed that this function of PKG-I indeed plays an important role in modulating calcium release from intracellular stores in nociceptive neurons of the DRG. We performed Fura-2-based calcium imaging on dissociated DRG neurons derived from PKG-I^fl/fl^ and SNS-PKG-I^−/−^ mice using Fluro488-conjugated Isolectin B_4_ (IB_4_-Fluro488) for live identification of small-diameter nociceptive neurons (neurons dually labelled with Fura-2 and IB4-Fluor488 are indicated by arrowheads in Figure 6C). The baseline values of the Fura2 ratios (F340/F380) were not significantly different between control mice (1.049±0.010) and SNS-PKG-I^−/−^ mice (1.040±0.008) (*p*>0.05; Student's *t* test). Stimulation of calcium release via activation of G_q/11_-phospholipase C-IP_3_R1 pathway by addition of ligands, such as bradykinin (BK) and the P2Y-receptor ligand, UTP, led to typical increases in the ratio of Fura-2 fluorescence at 340/380 nm in neurons from PKG-I^fl/fl^ mice, which were markedly reduced in neurons from SNS-PKG-I^−/−^ mice (see Figure 6D for typical examples and Figure 6E for quantitative summary; *p*<0.001 with respect to BK and UTP). In contrast, Fura2-labelled neurons with large-diameter somata, which were IB4-negative, did not show differences in calcium responses between PKG-I^fl/fl^ mice and SNS-PKG-I^−/−^ mice (an example is indicated by arrow in Figure 6C and quantitative summary is given in Figure 6E). In contrast, rapid calcium influx caused by KCl-induced depolarisation or capsaicin-evoked influx of calcium via TRPV channels was comparable in DRG neurons derived from SNS-PKG-I^−/−^ mice and PKG-I^fl/fl^ mice (Figure 6D,E; *p*>0.05), showing thereby that a loss of PKG-I in nociceptive neurons is specifically linked to defects in IP_3_R-mediated calcium release from intracellular stores.

Taken together, these biochemical and functional experiments suggest that following persistent nociceptive stimulation, PKG-I mediates potentiation of IP_3_R1 activity and MLC phosphorylation in sensory neurons, which is functionally linked to synaptic LTP at synapses between C-nociceptors and spinal-PAG projection neurons.

### A Key Functional Role for PKG-I and Its Substrates in a Behavioural Paradigm for Spinal Sensitization in Vivo

**Figure 7:**
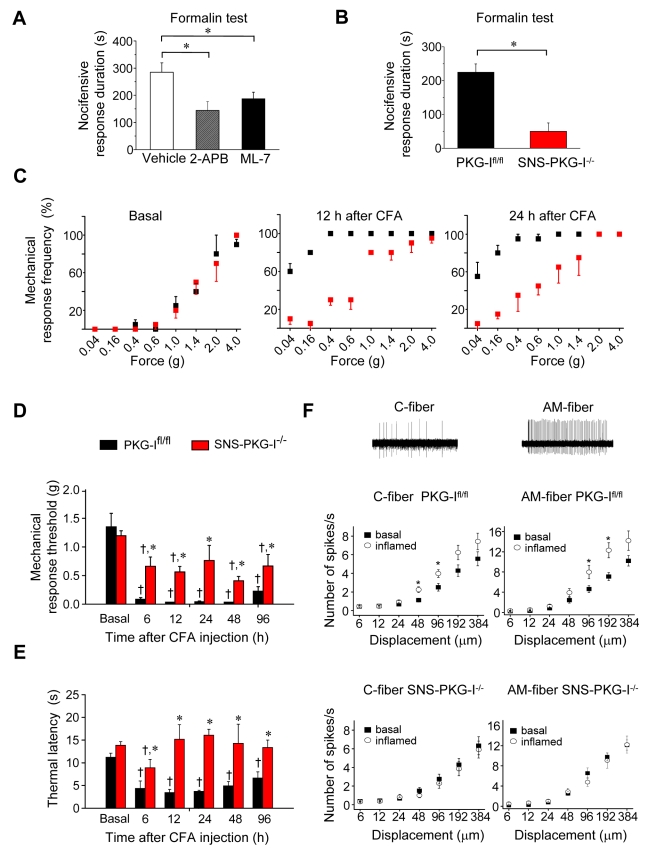
Presynaptic PKG-I and molecular mediators of spinal synaptic potentiation are required for behavioural manifestation of nociceptive hypersensitivity. (A, B) Nociceptive hypersensitivity in the intraplantar formalin test (phase II) is significantly reduced upon intrathecal delivery of an IP_3_R blocker (2-APB) or an inhibitor of MLC phosphorylation (ML-7) in wild-type mice (A). SNS-PKG-I^−/−^ mice show significantly reduced formalin responses than PKG-I^fl/fl^ littermates (B). (C) Comparison of response frequency to von Frey hairs in SNS-PKG-I^−/−^ mice (*n* = 10; red squares) and PKG-I^fl/fl^ mice (*n* = 10; black squares) prior to and at 12 h and 24 h following unilateral hindpaw inflammation caused by intraplantar injection of Complete Freund's adjuvant (CFA). Note that leftward deviations in stimulus-response curves in the inflamed state over the basal state are much less pronounced in SNS-PKG-I^−/−^ mice as compared with their control PKG-I^fl/fl^ littermates. (D, E) Magnitude and time-course of mechanical hypersensitivity to plantar von Frey hair application (panel D) and thermal hyperalgesia to radiant heat (panel E) following unilateral hindpaw inflammation caused by intraplantar injection of Complete Freund's adjuvant (CFA) to SNS-PKG-I^−/−^ and PKG-I^fl/fl^ mice are shown. * *p*<0.05 as compared to PKG-I^fl/fl^ mice, † *p*<0.05 in PKG-I^fl/fl^ mice or in SNS-PKG-I^−/−^ mice as compared to their own basal values, and ANOVA of random measures followed by post hoc Fisher's test. (F) Analysis of excitability of peripheral nociceptors in PKG-I^fl/fl^ mice and SNS-PKG-I^−/−^ mice. Electrophysiological recordings from C-mechanoceptors (*n* = 49) and A-δ-type mechanoceptors (AM, *n* = 43) in the paw skin-saphenous nerve preparation derived from the paws of PKG-I^fl/fl^ mice revealed an inflammation-induced increase in the frequency of firing evoked by application of pressure via a nanomotor (expressed in terms of displacement). * indicates significant statistical difference (*p*<0.05, ANOVA, post hoc Fisher's test). Similar recordings in inflamed paw skin derived from SNS-PKG-I^−/−^ mice showed no significant changes between response properties of C-fibers (*n* = 43 fibers) and AM-fibers as compared to control SNS-PKG-I^−/−^ mice (*n* = 43 fibers).

We then went on to address whether these findings bear relevance to pain-related behaviour in vivo and found a functional role for PKG-I and its substrates in behavioural paradigms for spinal sensitization. As a test system, we studied the Phase II of formalin-induced nocifensive behavioural responses, which are manifest at 10–60 min after intraplantar formalin injection, for two reasons: one, this represents a widely used paradigm for studying central changes in pain processing caused by a persistent activation of nociceptors [31], and two, intraplantar formalin induces synaptic LTP on spinal projection neurons with a matching time-course [5]. Formalin-induced phase II responses were significantly reduced upon intrathecal pretreatment with 2-APB or ML-7 to the lumbar spinal cord (Figure 7A; *p*<0.01 for 2-APB and ML-7 in comparison to vehicle control, respectively), implicating involvement of IP_3_R function and MLC phosphorylation, respectively. Similarly, SNS-PKG-I^−/−^ mice showed markedly reduced phase II responses than PKG-I^fl/fl^ mice (Figure 7B; *p*<0.001 as compared to PKG-I^fl/fl^ mice).

### Preserved Basal Nociception and Reduction of Primary Hyperalgesia in SNS-PKG-I^−/−^ Mice

Basal withdrawal thresholds and response latencies to acute application of paw pressure (e.g., as tested with a dynamic aesthesiometer) (Figure S6A, left panel) or thermal stimuli (e.g., a radiant infrared heat ramp) (Figure S6A, right panel), respectively, to the paw surface were found to be similar across SNS-PKG-I^−/−^ mice and their control littermates (*p*>0.05). Furthermore, motor performance on a Rotarod was unaffected in SNS-PKG-I^−/−^ mice (Figure S6B; *p* = 0.20). We have previously shown in details that SNS-Cre mice show no alterations in the processing of acute pain or chronic inflammatory or neuropathic pain [6],[32].

In the context of studying disease-induced pain hypersensitivity, we first focussed on a model of inflammatory pain which is associated with primary hyperalgesia in the inflamed area and ongoing nociceptive inputs from the periphery throughout the time of testing, namely unilateral hindpaw inflammation induced by injection of Complete Freund's Adjuvant (CFA) [32],[33]. CFA injection produced similar levels of edema in SNS-PKG-I^−/−^ and PKG-I^fl/fl^ mice (Figure S6C) and hypersensitivity to graded von Frey mechanical stimuli (Figure 7C,D) or to plantar heat (Figure 7E) applied to the ipsilateral paw was assessed at 6, 12, 24, 48, and 96 h thereafter. Following inflammation, PKG-I^fl/fl^ mice demonstrated the characteristic leftward and upward shift in the stimulus-response curve over basal curves reflecting mechanical hypersensitivity (black squares in Figure 7C). In contrast, SNS-PKG-I^−/−^ mice demonstrated a less marked deviation from baseline behaviour upon CFA-induced inflammation (red squares in Figure 7C). Furthermore, the relative drop in response thresholds to von Frey hairs (defined here as minimum force required to elicit 40% response frequency) in the inflamed state over basal (pre-CFA) state occurred to a significantly lesser extent in SNS-PKG-I^−/−^ mice as compared to PKG-I^fl/fl^ mice (left panel in Figure 7D; *p*<0.05 at all time points tested). Finally, SNS-PKG-I^−/−^ mice showed a significantly lower magnitude of thermal hyperalgesia than PKG-I^fl/fl^ mice at 6 h after CFA and did not show hyperalgesia at all from 12 h onwards after CFA injection, whereas PKG-I^fl/fl^ mice continued to show thermal hyperalgesia all the way up to the latest time point tested, namely 96 h following CFA injection (Figure 7E; *p*<0.01 between PKG-I^fl/fl^ and SNS-PKG-I^−/−^ mice at all time points tested). We infer from the above that the development of primary hyperalgesia and mechanical allodynia following somatic inflammation is impaired by a loss of PKG-I in nociceptors.

Although perturbation of spinal LTP may have contributed to the above phenotype in SNS-PKG-I^−/−^ mice, it is conceivable that a peripheral role for PKG-I in nociceptors may at least partially account for changes in primary hyperalgesia. To address functional changes in nociceptor sensitivity in the inflamed tissue, we utilised the skin-nerve preparation [34] to study the electrophysiological properties of identified polymodal C-fibres and Aδ-mechanoceptors (AM) in the saphenous nerve. The excitability of mechanoreceptive C-fibers and AM-fibers showed a small, but significant, increase following paw inflammation in PKG-I^fl/fl^ mice (see Figure 7F for typical examples), but not in SNS-PKG-I^−/−^ mice (Figure 7F). These data indicate defects in the development of peripheral sensitization in nociceptors of SNS-PKG-I^−/−^ mice, which could contribute to a reduction in primary hyperalgesia; however, they are unlikely to account for the marked defects in mechanical allodynia observed following inflammation in SNS-PKG-I^−/−^ mice.

### Models of Subacute and Chronic Central Hypersensitivity, Which Are Independent of Ongoing Input from Peripheral Nociceptors

**Figure 8:**
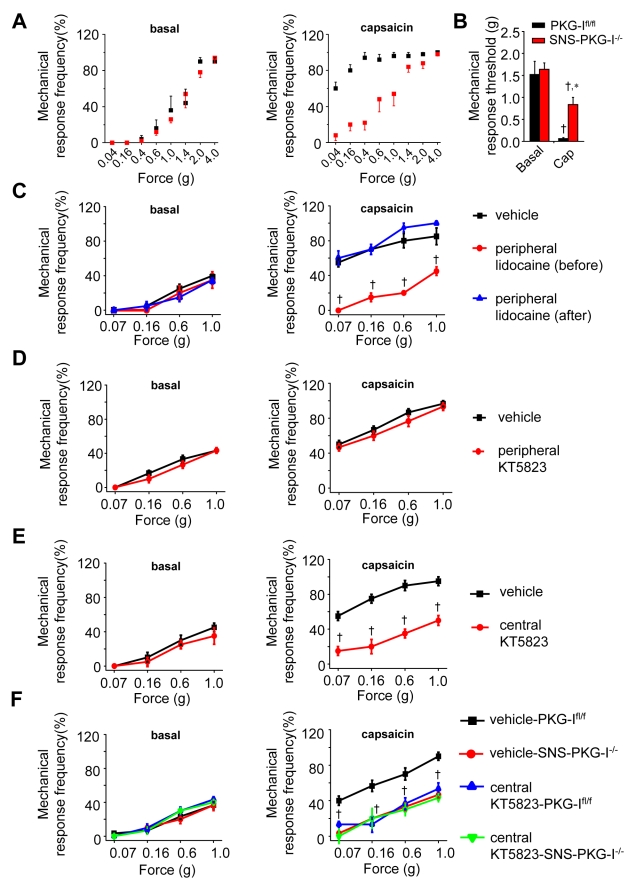
Development of secondary mechanical hypersensitivity evoked by capsaicin is reduced upon a nociceptor-specific loss of PKG-I via a centrally mediated mechanism involving presynaptic PKG-I. (A, B) Secondary hyperalgesia and allodynia to von Frey hairs applied to the plantar surface of the paw, which lies outside of the flare evoked by capsaicin injected into the flank, develop to a much lesser extent in SNS-PKG-I^−/−^ mice (red squares, *n* = 6) than in PKG-I^fl/fl^ mice (black squares, *n* = 6). Shown are stimulus-response curves (A) and the summary of response thresholds to von Frey hair application to the paw prior to and at 15 min following capsaicin injection (B). All data points represent mean ± S.E.M. † and * indicate significant statistical difference as compared to basal state or PKG-I^fl/fl^ mice, respectively. (C) Peripheral nerve blockade with lidocaine inhibits capsaicin-induced mechanical hypersensitivity in wild-type mice when lidocaine is injected prior to capsaicin, but not when lidocaine is injected at 15 min after capsaicin, indicating that capsaicin-induced mechanical hypersensitivity is independent of on-going input from peripheral nociceptors; *n* = 6 mice per group. (D, E) Pharmacological inhibition of PKG-I with KT5823 in wild-type mice inhibits capsaicin-induced mechanical hypersensitivity when KT5823 is injected intrathecally (panel E), but not when it was injected peripherally (panel D), prior to capsaicin indicating a role for central, but not peripherally expressed, PKG-I; *n* = 6 mice per group. (F) Inhibition of capsaicin-induced mechanical hypersensitivity by intrathecally injected KT5823 comes about in PKG-I^fl/fl^ mice, but not in SNS-PKG-I^−/−^ mice, indicating a specific involvement of PKG-I expressed in central nociceptor terminals; *n* = 6 mice per group. * *p*<0.05 as compared to the vehicle group in panels C–E and vehicle-treated PKG-I^fl/fl^ mice in panel F. ANOVA, followed by post hoc Fisher's test.

To explore central contributions, we utilised two models of aberrant pain which are triggered initially by peripheral inputs but do not require ongoing nociceptor activity in the periphery for maintenance. For example, capsaicin injection in the skin activates C-fibers and evokes hyperalgesia in the area of the flare (primary hyperalegsia) as well as outside of the flare (secondary hyperalgesia). In PKG-I^fl/fl^ mice, we observed that injection of capsaicin in the skin of the lower thigh produced a marked allodynia at the hindpaw plantar surface, which was clearly excluded from the area capsaicin-induced flare (see shift in von Frey response frequency in Figure 8A; black symbols). SNS-PGK-I^−/−^ mice showed markedly reduced secondary hypersensitivity with capsaicin as compared to PKG-I^fl/fl^ mice (red symbols in Figure 8A). Moreover, a capsaicin-induced drop in mechanical threshold (allodynia) was markedly reduced in SNS-PGK-I^−/−^ mice as compared to PKG-I^fl/fl^ mice (Figure 8B). It is well accepted that capsaicin-induced secondary mechanical hypersensitivity reflects C-fiber-evoked central amplification processes and can last for several hours, long after nociceptor responses to capsaicin have ceased owing to desensitisation of TRP channels [35]. Nevertheless, to rule out a potential contribution of ongoing peripheral inputs to the above-described phenotypic differences, we performed experiments in which nerve conduction was blocked with lidocaine in the peripheral dermatome in which capsaicin was injected in wild-type mice. As expected, lidocaine-induced nerve blockade prior to capsaicin injection blocked the induction of capsaicin-induced mechanical hypersensitivity (Figure 8C); in contrast, when lidocaine was injected 15 min after capsaicin, mechanical hypersensitivity developed normally (Figure 8C), indicating that beyond the initial trigger, capsaicin-induced mechanical hypersensitivity is independent of ongoing input from peripheral nociceptors. In further experiments, we addressed the peripheral and central contributions of PKG-I. Pharmacological inhibition of PKG-I with KT5823 injected prior to injection of capsaicin in the same dermatome in wild-type mice did not block the development of capsaicin-induced mechanical hypersensitivity (Figure 8D); in contrast, when KT5823 was injected intrathecally prior to peripheral capsaicin injection, the induction of mechanical hypersensitivity was markedly inhibited (Figure 8E), indicating a role for central PKG-I, but not peripherally expressed PKG-I. To further delineate the origin of the central (spinal) locus of PKG-I function, we undertook similar experiments in PKG-I^fl/fl^ mice and SNS-PGK-I^−/−^ mice. Interestingly, intrathecally administered PKG-I inhibitor blocked the development of capsaicin-induced mechanical hypersensitivity in PKG-I^fl/fl^ mice and did not lower mechanical sensitivity any further in SNS-PGK-I^−/−^ mice (Figure 8F), demonstrating thereby the presynaptic locus of its action.

**Figure 9:**
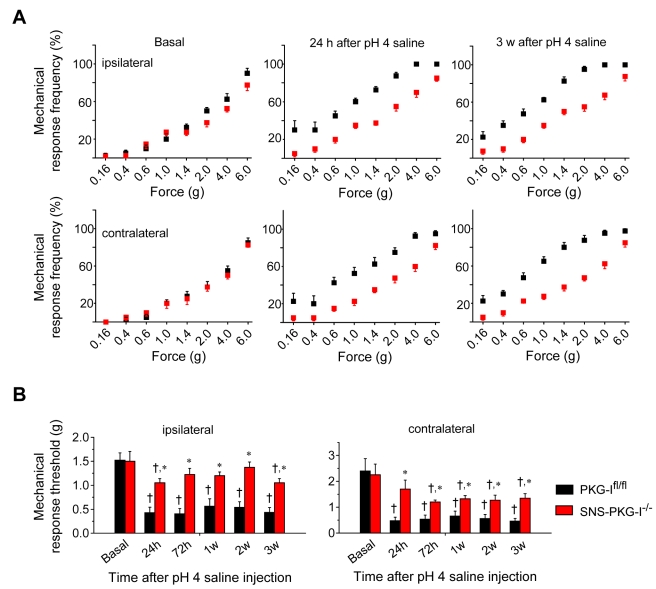
Centrally mediated bilateral plantar hypersensitivity in a model of chronic muscle pain which is independent of continuous peripheral input, is reduced upon a nociceptor-specific loss of PKG-I. (A) Increase in response frequency to plantar application of von Frey filaments at 24 h or 3 wk (3 w) following injection of acidic saline in the flank muscle is significantly more in PKG-I^fl/fl^ mice (*n* = 7; black squares) than in SNS-PKG-I^−/−^ mice (*n* = 8; red squares) in paws both ipsilateral and contralateral to the injected flank muscle. (B) Summary of response thresholds (defined as a force eliciting a response frequency of at least 40%) to von Frey hair application to the ipsilateral or contralateral paws, prior to and at 24 h, 72 h, and 1, 2, and 3 wk following acidic saline-induced muscle injury in SNS-PKG-I^−/−^ and PKG-I^fl/fl^ mice. All data points represent mean ± S.E.M. † and * indicate significant statistical difference (*p*<0.05, ANOVA, post hoc Fisher's test) as compared to basal state or PKG-I^fl/fl^ mice, respectively.

In the muscle pain model by Sluka and colleagues [36], two consecutive injections of dilute acidic saline in the flank muscle lead to secondary mechanical hypersensitivity in the ipsilateral and contralateral paws, which lasts for several weeks. The initial peripheral insult (i.e., flank muscle) is spatially distinct from the area of application of nociceptive stimuli (paw surface), ruling out a contribution of peripheral paw sensitization to the behavioural phenotype. Secondly, it has been shown in details previously that the secondary hyperalgesia in the paw lasts for several months after muscle injection, is not associated with any persistent inflammation or injury to the muscle tissue, is independent of peripheral inputs, and is thus central in origin [36]. Upon testing at 24 h after the induction of muscle pain, PKG-I^fl/fl^ mice demonstrated a pronounced leftward and upward shift in the stimulus-response curve to von Frey hairs applied to the plantar paw surface (black squares in Figure 9A, middle panel), which was still evident 3 wk later in the ipsilateral (Figure 9A, right panel); this changes come about in the paw ipsilateral to the injected flank muscle (upper panels in Figure 9A) as well as in the contralateral paw (lower panels in Figure 9A). In contrast, SNS-PKG-I^−/−^ mice did not show significant deviations (red squares in Figure 9A). Analysis of paw-withdrawal thresholds to graded pressure also consistently revealed that muscle injection-induced drop in paw mechanical thresholds at the paws was significantly lesser in SNS-PKG-I^−/−^ mice than in control littermates at all time points tested (Figure 9B). In conclusion, these analyses support an essential role for presynaptic PKG-I in nociceptor terminals in central mechanisms of secondary mechanical hypersensitivity.

### Signalling Pathways Upstream of PKG-I Activation in Spinal Nociceptive Circuits

**Figure 10:**
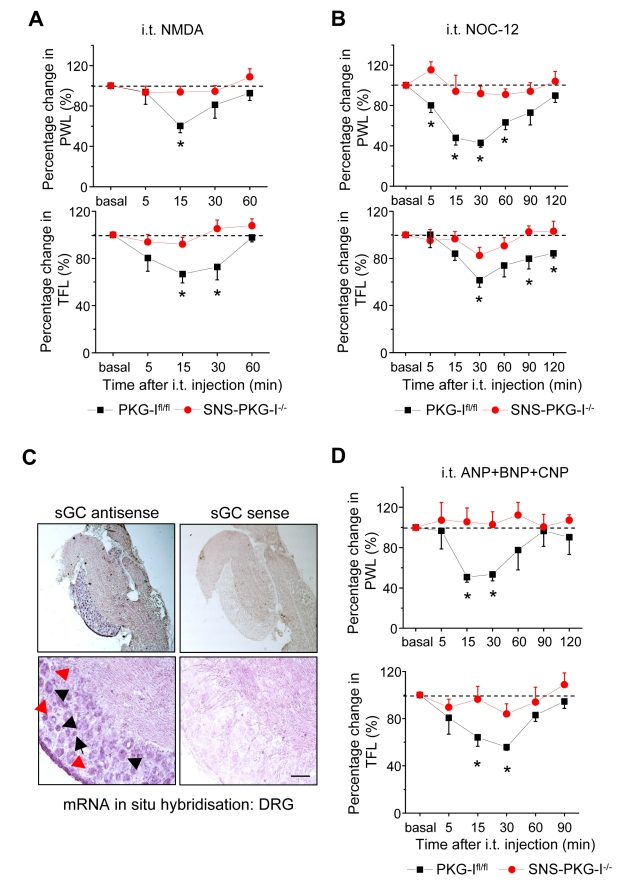
Requirement of presynaptic PKG-I for spinal facilitation of nociception induced by the NMDA-NO-cGMP pathway and the natriuretic peptide-cGMP pathway. (A, B) Facilitation of latency to paw withdrawal (PWL) or tail-flick latency (TFL) following intrathecal application of NMDA (100 fmol), the NO-donor or NOC-12 (17 nmol) in PKG-I^fl/fl^ mice (black squares), but not in SNS-PKG-I^−/−^ mice (red circles). (C) mRNA in situ hybridisation showing expression of the catalytic subunit of soluble guanylate cyclise (sGC) in populations of small-diameter neurons (black arrows), large-diameter neurons (black arrowheads), and satellite cells (red arrows) in mouse DRG sections. Scale bar represents 50 µm. (D) Facilitation of latency to paw withdrawal or tail-flick following intrathecal application of a natriuretic peptide cocktail (330 pmol each of ANP, BNP, CNP) in PKG-I^fl/fl^ mice (black squares), but not in SNS-PKG-I^−/−^ mice (red circles). All data points represent mean ± S.E.M. * indicates significant statistical difference (*p*<0.05, ANOVA, post hoc Fisher's test) as compared to PKG-I^fl/fl^ mice, respectively. In all experiments, *n* = 8–10 mice per genotype or treatment group.

Finally, we asked whether PKG-I expressed in nociceptors constitutes an important target of the NMDA-NOS-cGMP pathway. Consistent with previous studies [37], intrathecally administered NMDA produced a rapid facilitation of the paw withdrawal reflex (Figure 10A). Importantly, in striking contrast to PKG-I^fl/fl^ mice (black symbols), SNS-PKG-I^−/−^ mice completely failed to develop hyperalgesia following intrathecal NMDA delivery (red symbols, upper panel in Figure 10A). Similar results were obtained upon delivery of an NO donor, NOC-12, to the spinal cord via intrathecal catheters (upper panel in Figure 10B). Furthermore, intrathecal delivery of NMDA and NOC-12 produced a facilitation of the tail flick reflex in PKG-I^fl/fl^ mice, but not in SNS-PKG-I^−/−^ mice (lower panels in Figure 10A,B), showing thereby that PKG-I is critically required for the pro-nociceptive functions of the NMDA and NO.

Because soluble guanylyl cyclases (sGC) represent a key molecular link between NO and activation of PKG-I, the above results imply that NO activates sGC in spinal presynaptic terminals of nociceptors. While some studies report a lack of sGC expression in DRG neurons [38], others reported expression in a population of small diameter DRG neurons and in primary afferents [39],[40]. Here, we carried out mRNA in situ hybridisation using riboprobes recognising the beta subunit of sGC on mouse DRG sections and observed distinct, specific signals over the soma of several large and small-diameter DRG neurons (arrowheads and arrows in Figure 10C, respectively). Furthermore, the satellite cells surrounding DRG neurons showed dense signals (red arrows in Figure 10C). Sense control probes did not yield any appreciable signals (Figure 10C). These results indicate that sGC mRNA is expressed in sensory neurons of the DRG.

In addition to sGC enzymes, which are directly activated upon NO, the membrane-bound guanylyl cyclases (mGC; Npr family) also contribute to cGMP production in some organs (e.g., in the cardiovascular system) [41]. Stimulated by recent reports on expression of mGCs in DRG neurons [42], we administered a cocktail of natriuretic peptides (ANP, BNP, and CNP) intrathecally and observed marked hyperalgesia within 15 min after delivery, which lasted for about 45–50 min in wild-type mice (unpublished data) and PKG-I^fl/fl^ mice (Figure 10D). Interestingly, natriuretic peptide-induced hyperalgesia was also entirely abrogated in SNS-PKG-I^−/−^ mice (Figure 10D). These results suggest that the NMDA-NOS-soluble guanylyl cyclase-cGMP pathway as well as the natriuretic peptide-mGC-cGMP pathways converge upon PKG-I expressed in spinal terminals of nociceptors to modulate nociceptive processing in the spinal cord.

### Reinstating PKG-I Expression in DRG Neurons of SNS-PKG-I^−/−^ Mice Restores Mechanical Hypersensitivity

**Figure 11:**
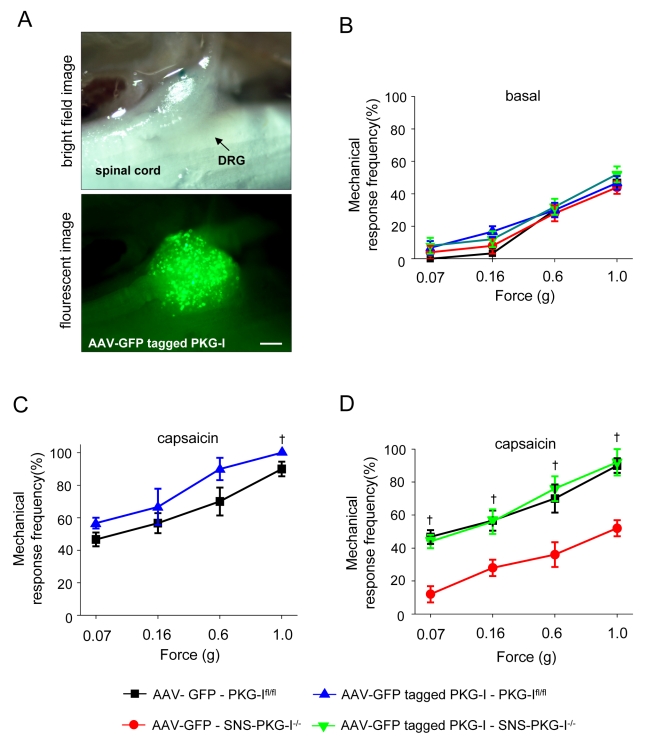
Rescue of defects in plasticity mechanisms in SNS-PKG-I^−/−^ mice by viral expression of GFP-tagged PKG-I specifically in the L3-L4 dorsal root ganglia (DRG) of adult mice. (A) Photomicrographs showing expression of GFP-tagged PKG-I in the L4 DRG upon delivery via adeno-associated virions (AAV). Scale bar represents 250 µm. (B) Basal mechanical sensitivity to graded mechanical stimuli (von Frey) is unchanged upon overexpression of GFP-tagged PKG-I or GFP alone (control) in DRG neurons of PKG-I^fl/fl^ mice and SNS-PKG-I^−/−^ mice. (C) PKG-I^fl/fl^ mice expressing GFP-tagged PKG-I show a minor increase in magnitude of capsaicin-induced mechanical hypersensitivity than PKG-I^fl/fl^ mice expressing GFP. † *p*<0.05 as compared to AAV-GFP-PKG-I^fl/fl^ mice. (D) SNS-PKG-I^−/−^ mice overexpressing GFP in DRG show markedly reduced mechanical hypersensitivity with capsaicin than PKG-I^fl/fl^ mice overexpressing GFP. Overexpression of GFP-tagged PKG-I fully restored mechanical hypersensitivity in SNS-PKG-I^−/−^ mice. † *p*<0.05 indicates significant differences in the AAV-GFP-SNS-PKG-I^−/−^ mice as compared to the other two groups. ANOVA followed by post hoc Fisher's test.

Finally, we undertook experiments to test whether PKG-I expression alone or some downstream factor perturbed by an early loss of PKG-I is responsible for the deficits in pain hypersensitivity in SNS-PKG-I^−/−^ mice. We constructed chimeric Adeno-associated virions of the serotypes AAV1 and AAV2 expressing an C-terminally GFP-tagged version of the murine PKG-I cDNA [43]. Injection in unilateral L3 and L4 DRGs in vivo led to a broad expression in the DRG. AAV1/2 chimeric virions expressing GFP alone served as controls. PKG-I^fl/fl^ mice and SNS-PKG-I^−/−^ mice expressing GFP-tagged PKG-I or GFP alone showed normal basal sensitivity to graded von Frey stimuli (Figure 11B). Upon peripheral injection of capsaicin, PKG-I^fl/fl^ mice expressing GFP-tagged PKG-I showed a small increase in mechanical hypersensitivity than PKG-I^fl/fl^ mice expressing GFP, which was only statistically significant at some intensities of mechanical stimuli (Figure 11C). As expected, SNS-PKG-I^−/−^ mice overexpressing GFP in DRG showed markedly reduced mechanical hypersensitivity with capsaicin than PKG-I^fl/fl^ mice overexpressing GFP. Importantly, overexpression of GFP-tagged PKG-I fully restored mechanical hypersensitivity in SNS-PKG-I^−/−^ mice (Figure 11D). This indicates that expression of PKG-I is both necessary and sufficient for inducing centrally maintained hypersensitivity upon persistent peripheral activation of C-fibers.

## Discussion

In contrast to the intensively studied forms of LTP in the hippocampus, very few studies have addressed cellular and molecular mechanisms of LTP at spinal synapses regulating the flow of nociceptive information from the periphery towards the brain [7],[24]. Here we observed that nociceptive activity-driven LTP at synapses between nociceptive terminals and spinal neurons projecting nociceptive inputs to the PAG requires presynaptic mechanisms for its full expression. Furthermore, our results indicate that this function is mediated by cGMP acting via PKG-I. We base our inferences on three main observations: (1) A specific loss of PKG-I in presynaptic, but not post-synaptic, compartments of this synapse abolished C-fiber-evoked LTP without altering basal neurotransmission at this synapse; (2) LTP was temporally accompanied by a decrease in the rate of synaptic failures in a presynaptic-PKG-I-dependent manner; and (3) LTP was associated with a change in the PPR, which did not take place when PKG-I was deleted presynaptically in nociceptor terminals. Importantly, higher magnitudes of LTP were consistently associated with a decrease in PPF, and thereby with an increase in release probability.

Previous studies have shown that the NMDA receptor-NO-cGMP pathway is important in the induction of spinal LTP [4],[5], and it has been assumed that this pathway comes into play in the post-synaptic compartment. However, all of the above signal transducers are also expressed presynaptically in afferent terminals in the spinal dorsal horn [44]. Thus, pre- and post-synaptic contributions to spinal LTP have not been worked out so far. There is evidence for a requirement for post-synaptic Ca^2+^ change for the induction of the LTP (i.e., in experiments with BAPTA in recording pipette; [5]). Taken together with our results, this suggests that a calcium-dependent postsynaptic mechanism may be required for the *induction* of LTP (e.g., via NMDA receptor-dependent generation of NO); in contrast, a presynaptic change involving cGMP- and PKG-I-dependent increase in neurotransmitter release may mediate the *expression* of LTP at synapses between nociceptors and spinal-PAG projection neurons.

Mechanistically, this may come about via involvement of multiple phosphorylation targets of PKG-I. While some targets have been identified in heterologous systems, very little is known about the nature and functional role of PKG-I targets in vivo. Here, we identified and validated two primary targets in DRG neurons, namely the IP_3_R1 and MLC, and observed that PKG-I modulates intracellular calcium release as well as MLC phosphorylation differently in DRG neurons as compared to other biological systems, such as the smooth muscle. For example, in some biological systems, PKG-I has been reported to negatively modulate calcium signals via its interaction with IRAG [18]. However, IRAG interacts selectively with the beta-isoform of PKG-I, which is barely expressed in the DRG, but not with PKG-I-alpha, the predominant form found in DRG neurons [10]. Furthermore, our observations that PKG-I potentiates calcium release induced by typical mediators of nociceptive sensitization, such as bradykinin, in identified nociceptive neurons and that repetitive activation of nociceptors in vivo leads to PKG-I-mediated phosphorylation of IP_3_R1 at serine 1755, a site associated with positive functional modulation, implicate PKG-I as a positive modulator of calcium signalling in nociceptive neurons. In light of electrophysiological analyses reported here, this raises the possibility that calcium released from IP_3_R1-gated stores may participate in modulating presynaptic function. Although a few studies at hippocampal synapses have proposed an involvement of calcium stores in modulation of presynaptic release [45],[46], underlying cellular mechanisms are not known. Results of this study suggest that activation of presynaptic PKG-I may constitute the molecular link between synaptic activity and the elevation of resting levels of calcium in presynaptic terminals, thereby potentiating synaptic transmission via an increase in release probability. Furthermore, we observed that MLC was phosphorylated in a nociceptive activity-dependent manner and that PKG-I is required for MLC phosphorylation in the DRG. Although our electrophysiological data implicate phosphorylated MLC in LTP at spinal synapses, not much can be inferred about downstream mechanisms at this stage. At central synapses, MLC phosphorylation was initially implicated in vesicle transport and in regulation of vesicular pools [47]; however, these inferences could not be corroborated in detailed subsequent analyses [48]. Taken together, more detailed analyses overcoming current technical hindrances in studying mobilization of vesicular pools in the complex circuitry of the DRG and spinal dorsal horn will be required to understand mechanisms underlying PKG-I-mediated modulation of release probability at this synapse.

The possibility of functionally linking synaptic changes described here to changes in nociceptive behaviour simultaneously represents a good opportunity and a major challenge. As the first parameter to test this relationship, we focused on the phase II responses in the intraplantar formalin test, which has been attributed to spinal nociceptive sensitization triggered by an initial barrage of C-fiber inputs [31]. Indeed, we observed that presynaptic loss of PKG-I as well as functional perturbation of MLCK and IP_3_R, its substrates involved in LTP, inhibited phase II behavioural responses. However, a contribution of central mechanisms could not be inferred from the formalin data due to several reasons: Although MLCK/IP_3_R inhibitors were administered spinally, the genetically induced loss of PKG-I in SNS mice occurred throughout the nociceptor, including peripheral terminals. Moreover, there is still some ongoing activation of peripheral nociceptors in the phase II of the formalin response [31]. Indeed, our electrophysiological analyses in the CFA inflammatory pain model suggested that peripheral PKG-I may contribute to primary hyperalgesia.

Therefore, we focused on pain models in which nociceptive hypersensitivity is triggered by peripheral nociceptors, but maintained via central mechanisms that outlast and are independent of peripheral inputs. One of these is the chronic muscle pain model in which injections of dilute acidic saline in the gastronemius muscle evokes a long-lasting secondary mechanical hyperalgesia in the ipsilateral and contralateral paws, which lasts for several weeks, is unrelated to muscle damage and is not maintained by continued primary afferent input from the site of injury, as shown by experiments involving dorsal rhizotomy and lidocaine injections in the muscle [36]. Similarly, we addressed capsaicin-induced secondary mechanical hyperalgesia outside of the primary flare, which albeit triggered by C-fiber inputs, is maintained via mechanisms of central origin as indicated by previous studies [35] and our analyses. In both models, we found marked defects in central hypersensitivity in SNS-PKG-I^−/−^ mice. Our results indicated that capsaicin-evoked mechanical hypersensitivity is neither dependent on peripheral PKG-I function nor does it require ongoing peripheral nociceptor sensitisation. Moreover, they revealed that PKG-I expressed in central terminals of nociceptors plays a decisive role in the induction of mechanical hypersensitivity after persistent C-fiber stimulation via capsaicin. Finally, reinstating PKG-I expression in the DRG in adult SNS-PKG mice fully restored capsaicin-evoked mechanical hypersensitivity, indicating that PKG-I directly, and not some factor affected by a genetic loss of PKG-I, is a functional determinant of C-fiber-evoked mechanical hypersensitivity.

In summary, this study shows that PKG-I expressed in nociceptors terminals is the principal target of cGMP at nociceptive synapses. Furthermore, it suggests that PKG-I-mediated presynaptic facilitation and LTP in spinal projection neurons is functionally involved in activity-dependent centrally mediated nociceptive hypersensitivity.

## Materials and Methods

Additional details on methods are provided in Text S1.

### Genetically Modified Mice

Homozygous mice carrying the flox allele of the mouse *prkg1* gene, which encodes the cGMP dependent kinase 1 (PKG-I^fl/fl^) [15], have been described previously in detail. PKG-I^fl/fl^ mice were crossed with SNS-Cre mice [16] to obtain PKG-I^fl/fl^;SNS-Cre^+^ mice (referred to as SNS-PKG-I^−/−^ mice in this article) and PKG-I^fl/fl^ mice (control littermates). Mice were crossed into the C57BL6 background for more than 8 generations. Mice lacking PKG-I globally (PKG-I^−/−^ mice) have been described before. Only littermates were used in all experiments to control for background effects.

### Spinal Cord Slice Preparation and Patch-Clamp Recordings

Mice (14–18 d old) were anesthetized with a mixture of Dormitor, Dormicum, and Fentanyl, and stereotactic injections of DiI into the PAG were carried out (see Text S1 for details). After 2 to 3 d, transverse 350–450 µm thick spinal cord slices with dorsal roots attached were obtained and whole cell patch clamp recordings of identified DiI-positive neurons were performed as described in Text S1. Test pulses of 0.1 ms with intensity of 3 mA were given at 30 s intervals to the dorsal root via a suction electrode. For studying the site of expression of synaptic potentiation, we used minimal stimulation in conditions of low release probability (in mM: NaCl 127; KCl, 1.8; KH_2_PO_4_, 1.2; Ca^2+^ 1.0; Mg^2+^, 5; NaHCO_3_, 26; glucose, 15; oxygenated with 95% O_2_, 5% CO_2_; pH 7.4). Dorsal root was stimulated at intensity of threshold to evoke EPSCs on DiI-labelled spino-PAG projection neuron. Under these conditions, the failure rate was 60.9%±6.3% (*n* = 10). To induce synaptic potentiation, low frequency stimulation (conditioning stimulus, 2 Hz for 2 min) was applied to dorsal root as a conditioning stimulus with the same intensity as the test stimulus [5]. The recording mode during conditioning stimulation was the same as that before and after conditioning stimulation. Neurons are voltage clamped at −70 mV. Because a suction electrode was utilized to stimulate the whole root, a suprathreshold stimulus was required to fully recruit C-fibers in the root [5]. Synaptic strength was quantified by assessing the peak amplitudes of EPSCs. The mean amplitude of 4–5 EPSCs evoked by test stimuli prior to conditioning stimulation served as a control. Significant changes from control were assessed by measuring the peak amplitudes of five consecutive EPSCs every 5 min after conditioning stimulation. Additional details are given in Text S1. In some experiments, blockers of inhibitory neurotransmission, such as Gabazine (10 µM) and Strychnine (1 µM), were added to the bath.

In a subset of animals, paired-pulse stimuli with an inter-stimulus interval of 110 ms (0.1 ms pulse duration, 3 mA intensity, every 30 s) were used (see Text S1 for details). Paired-pulse ratio (facilitation or depression) of C-fiber-evoked EPSC was calculated as the amplitude of the second C-eEPSC divided by that of the first C-eEPSC in a pair. In a subset of experiments, PKG-I inhibitors such as KT5823 (10 µM) or RKRARKE (250 µM) were infused post-synaptically via the patch pipette.

### Antibodies

The following antibodies were used for Western blots and biochemical analyses: anti-IP3R1, anti-_p_S^1755^ IP3R1 (kind gift from Prof. Richard Wojcikiewicz), anti-VASP (Alexis Biochemical), anti-MLC, anti-alpha tubulin (Sigma), anti-_p_Ser^239^ VASP, anti-_p_Thr^18^/_p_Ser^19^ MLC (Cell Signaling technology), anti-PKG-I antibody [18], secondary HRP labelled anti-rabbit (Sigma Aldrich), or anti-mouse (GK Healthcare UK Ltd.).

The following antibodies were used for immunohistochemistry: phospho-ERK1/2 antibody (Cell signalling), anti-Fos antibody (Chemicon), anti-Isolectin B4 antibody (vector laboratories), anti-Calcitonin gene related peptide antibody (Immunostar), anti-Neurofilament 200 antibody (Chemicon), anti-Substance P antibody (Chemicon), anti-PKG-I antibody [18], anti-PSD-95 antibody (a gift from M. Watanabe) and anti-TrkA antibody (a kind gift from Prof. L. F. Reichardt), and anti-cre antibody (Novagen).

### Application of Drugs in Vivo

The soluble guanylyl cyclase inhibitor, ODQ (25 mg/kg body weight; sigma Aldrich), and the membrane guanylyl cyclase inhibitor, LY83583 (12.5 mg/kg body weight; sigma Aldrich), were dissolved in 50% DMSO and injected in a volume of 250 µl intraperitoneally. The following drugs were administered intrathecally in vivo: an inhibitor of MLCK (ML-7; 15 nmol Alexis Biochemical, dissolved in 5% DMSO), an inhibitor of IP_3_R (2-APB; 2 nmol; Calbiochem), NMDA (100 fmol; Sigma Aldrich), the NO donor, NOC-12 (17 nmol; Sigma Aldrich), atrial natriuretic peptide, brain natriuretic peptide and c-type natriuretic peptide (rANP1-28, mBNP45, and hCNP1-22; 330 pmol of each natriuretic peptide; American peptide company, Inc., USA), and the PKG-I inhibitor KT5283 (200 pmoles). See Text S1 for details on intrathecal delivery. Mice were allowed to recover for 2 d after surgery, and only animals showing complete lack of motor abnormalities were used for further experiments. 5 µl of drugs were applied followed by flushing of the catheter with 10 µl of 0.9% saline. The following drugs were administered peripherally in the vicinity of the paw in experiments pertaining to capsaicin-induced mechanical hypersensitivity: KT5823 (200 pmoles) and lidocaine (10 µl of 2%).

### Skin-Nerve Preparation and Recordings from Peripheral Nociceptors

A total of 17 PKG-I^fl/fl^ and 15 SNS-PKG-I^−/−^ mice were used in the electrophysiological recordings of nerve activity. An ex vivo skin-nerve preparation was used to study the properties of mechanosensitive C- and A-δ afferent fibres which innervate the skin in the inflamed area 24 h following CFA inoculation (20 µl) as described previously (see Text S1 for details).

### Nociceptive Tests and Mouse Models of Pain

All animal use procedures were in accordance with ethical guidelines imposed by the local governing body (Regierungspräsidium Karlsruhe, Germany). All behavioural measurements were done in awake, unrestrained, age-matched mice of both sexes that were more than 3 mo old by individuals who were blinded to the genotype of the mice being analyzed (see Text S1 for details).

### Viral-Mediated Expression in DRG in Vivo

The open reading frame of mouse PKG-I fused C-terminally with GFP [43] or EGFP alone was cloned in an AAV expression construct, and chimeric AAV1/2 virions were generated using standard protocols. Virions were diluted 1∶2 with 20% mannitol and injected unilaterally into L3 and L4 DRGs (1 µl per DRG, or approx. 10^7^ transfection units per DRG) in deeply anesthetized mice as described in detail previously [49]. Mice were tested in behavioural tests 2 wk after injection. At the end of the experiment, mice were perfused as described above and expression of GFP was confirmed via fluorescence analysis.

### Data Analysis and Statistics

All data are presented as mean ± standard error of the mean (S.E.M.). For comparisons of multiple groups, analysis of variance (ANOVA) for random measures was performed followed by post hoc Fisher's test to determine statistically significant differences. When comparing two groups that were studied in parallel, Student's *t* test was employed. Unless otherwise specified, the *p* values shown in the figures and text are derived from ANOVA and post hoc Fisher's test. *p*<0.05 was considered significant.

## Supporting Information

Figure S1 Further evidence for specific deletion of PKG-I in nociceptors, but not in spinal neurons. (A) Anti-Cre immunohistochemistry on SNS-Cre mice demonstrating Cre expression in small-diameter neurons of the DRG, but not in spinal cord. Scale bars represent 50 µm for DRG and 100 µm for spinal cord. (B & C) Western blot analysis reveals intact expression of PKG-I in the spinal cord and brain and reduced expression in the DRG of SNS-PKG-I^−/−^ mice as compared to their PKG-I^fl/fl^ littermates. Typical examples (B) and quantitative summary (C) from three independent Western blot experiments; Tubulin expression serves as a control. * *p*<0.001, ANOVA, post hoc Fisher's test.
(PDF)

Figure S2 Further evidence for normal gross development of the sensorimotor circuitry in SNS-PKG-I^−/−^ mice. (A) Adult PKG-I^fl/fl^ mice and SNS-PKG-I^−/−^ mice show similar patterns of targeting nociceptors in the spinal cord (upper panels) and the skin (lower panels) as shown by binding to TRITC-labelled Isolectin-B_4_ (red) and immunoreactivity for Substance P (Sub P) or CGRP (green). Level of immunoreactivity for Sub P in spinal superficial laminae was similar across genotypes (see results). (B) The density of synaptic contacts between Sub P-containing nociceptor terminals and PSD-95-expressing postsnyptic spines on spinal neurons in the spinal superficial laminae is similar across genotypes. Quantitative values on the right indicate mean percentage of association of substance P and PSD-95. (C, D) Typical examples (C) and quantitative analysis of heat pain-induced internalization of a Sub P receptor via NK1R immunoreactivity in spinal dorsal horn neurons, which was comparable across genotypes. (E) Normal spinal laminar development in adult SNS-PKG-I^−/−^ mice and their PKG-I^fl/fl^ littermates. Cell bodies of spinal neurons were stained via anti-NeuN immunohistochemistry. Scale bars represent 100 µm in panel A and 2 µm in panel B. 15 µm in panel C and 100 µm in panel E.
(PDF)

Figure S3 Further validation of C-fiber-evoked synaptic long-term potentiation (LTP) at spinal synapses in wild-type mice. (A) Typical examples of retrogradely labelled spinal lamina I projection neurons (lower panels) following DiI injections into the PAG (upper panel). (B) LTP was preserved upon spinal blockade of inhibitory neurotransmission with a combination of bicuculline and strychnine; *n* = 12 slices each. (C) Blockade of PKG-I specifically in the postsynaptic neuron via application of KT5823 in the patch pipette did not affect LTP; *n* = 6 slices each. (D) Quantitative summary of the magnitude of increase in c-EPSCs in the above groups at 30 min after the conditioning stimulus (LFS) over basal vales (normalized to 100). RKRARKE represents a distinct PKG-I inhibitor, which was tested in parallel experiments.
(PDF)

Figure S4 Magnitude of LTP at synapses between C-fibers and spino-PAG neurons is plotted as a function of change in PPF or PPD at the same synapse in SNS-PKG-I^−/−^ mice and their PKG-I^fl/fl^ littermates. It is noteworthy that in PKG-I^fl/fl^ mice, synapses showing a large LTP show a decrease in PPF, indicating an increase in the release probability. An increase in PPF is only seen with a few synapses that do not show a robust LTP. These changes do not come about in SNS-PKG-I^−/−^ mice.
(PDF)

Figure S5 Nociceptive activity-driven phosphorylation of PKG-I substrates in DRG and spinal cord in vivo. (A, B) A typical example (A) and quantitative summary (B) of levels of VASP phosphorylated on serine 239 in L4-L5 DRGs of SNS-PKG-I^−/−^ mice and PKG-I^fl/fl^ littermates in the naïve state or following formalin injection in the hindpaws. (C) Expression levels of total VASP or MLC do not vary between the above experimental groups. (D) Expression levels of total IP_3_R1 do not vary between the above experimental groups. In all panels, quantitative summaries are derived from three such independent experiments, each involving eight mice/group. * *p*<0.05, ANOVA of random measures followed by post hoc Fisher's test. Data are represented as mean ± S.E.M.
(PDF)

Figure S6 Nociceptive withdrawal responses and development of paw inflammation in SNS-PKG-I^−/−^ mice and their PKG-I^fl/fl^ littermates. (A) In comparison with PKG-I^fl/fl^ mice (*n* = 8), SNS-PKG-I^−/−^ mice (*n* = 8) show comparable paw withdrawal latency (PWL; *p* = 0.26) to radiant heat and paw withdrawal threshold (PWT; *p* = 0.24) to punctuate pressure. (B) Latency to fall from a rotating rod was similar in SNS-PKG-I^−/−^ mice (*n* = 6) and PKG-I^fl/fl^ mice (*n* = 6). (C) The magnitude of paw edema was similar between SNS-PKG-I^−/−^ mice and their PKG-I^fl/fl^ littermates following CFA-induced paw inflammation. Shown are complementary analyses of paw size in terms of paw volume (in cubic mm; length×breadth×height) and volume of water displaced by paws (in ml). All data points represent mean ± S.E.M. *n* = 8–10 mice per genotype or treatment group.
(PDF)

Text S1 Supplementary Methods and Materials.
(DOCX)

## Abbreviations

AHP: after hyperpolarisation
BK: bradykinin
CFA: Complete Freund's Adjuvant
CNG: cyclic nucleotide-gated
DRG: dorsal root ganglia
HCN: hyperpolarization-activated cyclic nucleotide-gated
LTP: long-term potentiation
mGC: membrane-bound guanylyl cyclases
MLC: myosin light chains
PAG: periaqueductal grey
PDEs: phosphodiesterases
PKG-I: Protein Kinase G1
PPD: paired-pulse depression
PPF: paired-pulse facilitation
PPR: paired-pulse ratio
sGC: soluble guanylyl cyclases
VASP: Vasodilator-stimulated phosphoprotein
