# Supplementary figures and images for: Presynaptically Localized Cyclic GMP-Dependent Protein Kinase 1 Is a Key Determinant of Spinal Synaptic Potentiation and Pain Hypersensitivity
Source: PLoS Biol. 2012 Mar 13;10(3):e1001283. doi: 10.1371/journal.pbio.1001283 (PMC3302842; doi:10.1371/journal.pbio.1001283)

## Supplementary figure - 1

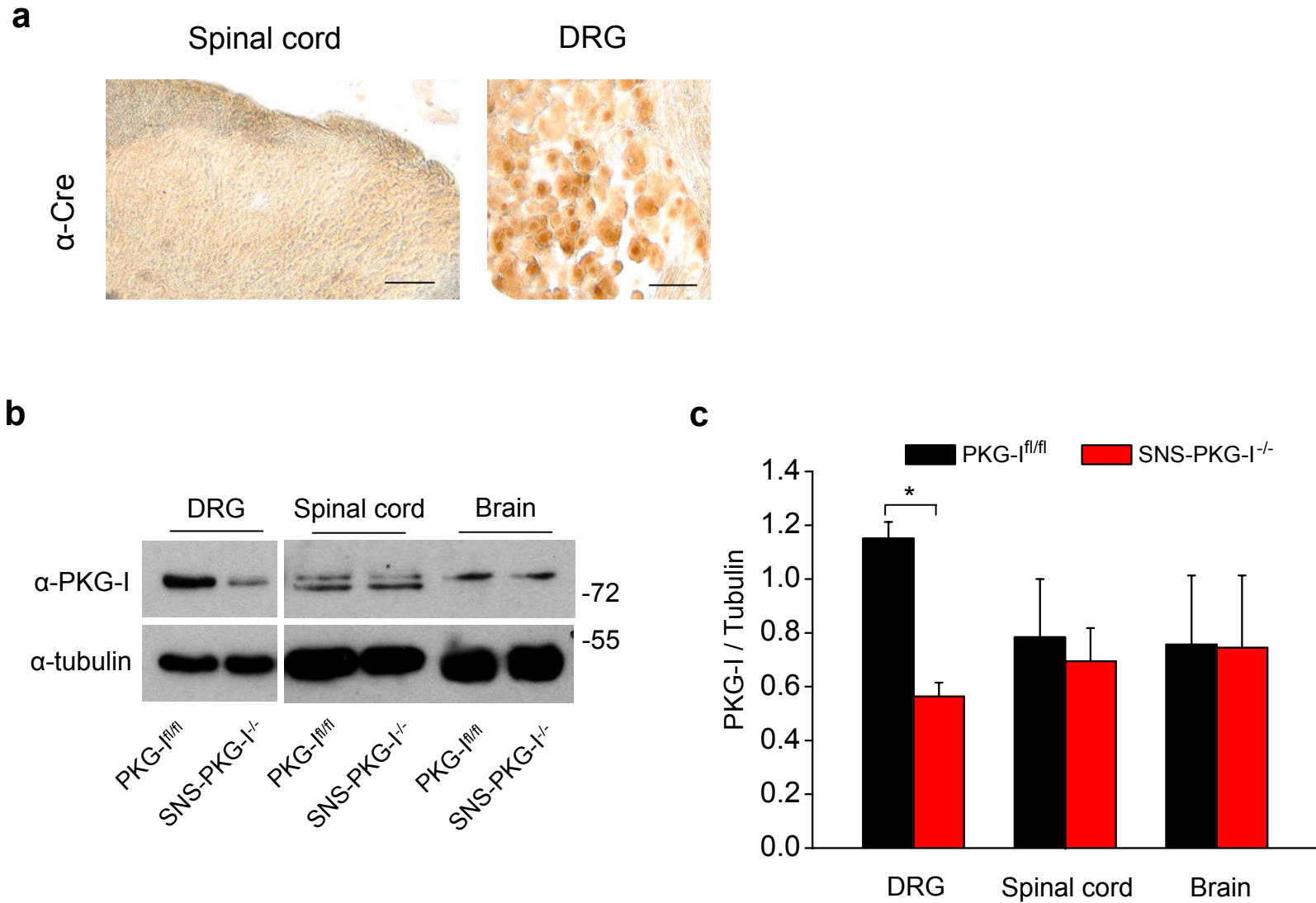

Supplement: Figure S1 — Further evidence for specific deletion of PKG-I in nociceptors, but not in spinal neurons. (A) Anti-Cre immunohistochemistry on SNS-Cre mice demonstrating Cre expression in small-diameter neurons of the DRG, but not in spinal cord. Scale bars represent 50 µm for DRG and 100 µm for spinal cord. (B & C) Western blot analysis reveals intact expression of PKG-I in the spinal cord and brain and reduced expression in the DRG of SNS-PKG-I−/− mice as compared to their PKG-Ifl/fl littermates. Typical examples (B) and quantitative summary (C) from three independent Western blot experiments; Tubulin expression serves as a control. * p<0.001, ANOVA, post hoc Fisher's test. (PDF) [file pbio.1001283.s001.pdf]

Supplementary Fig - 3

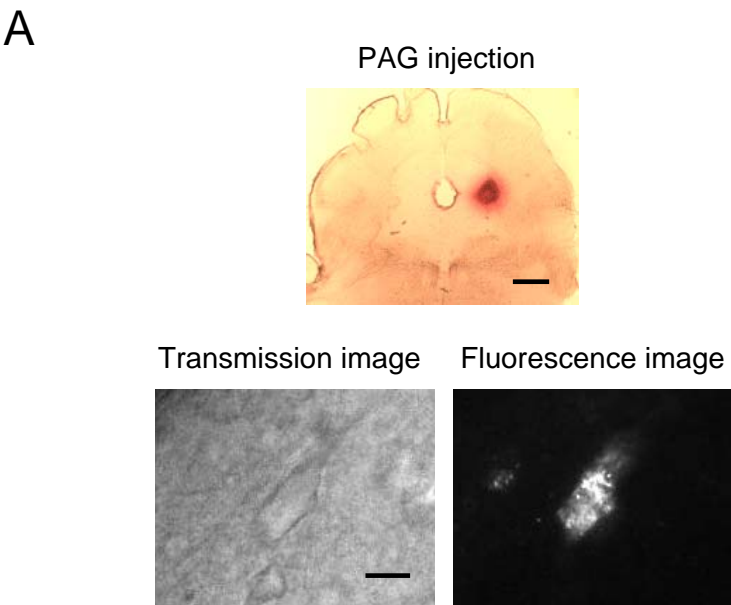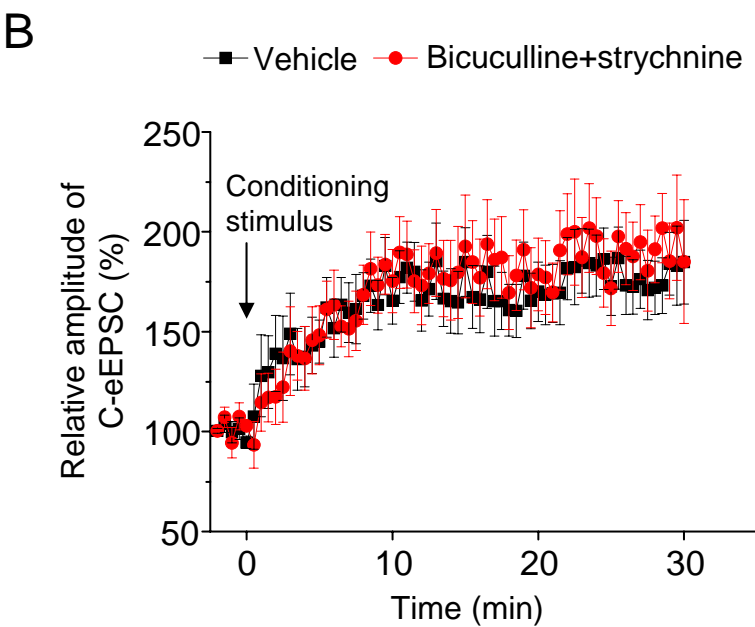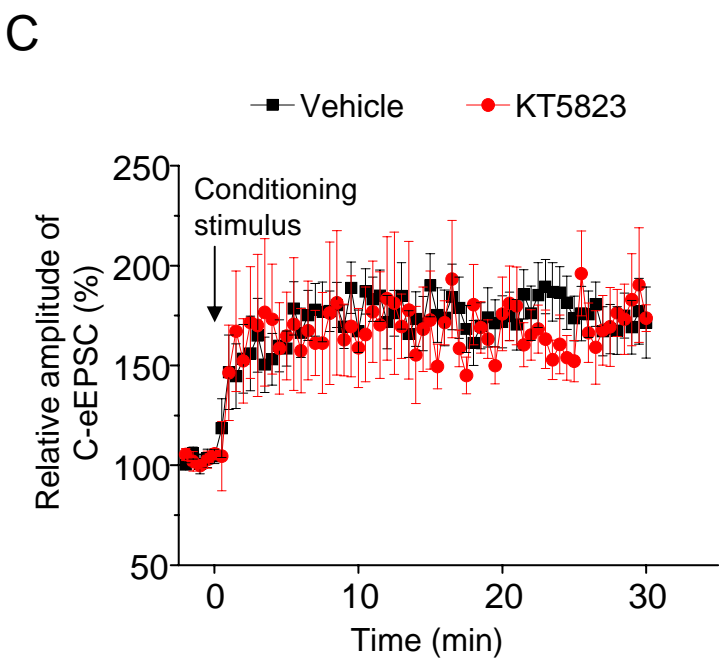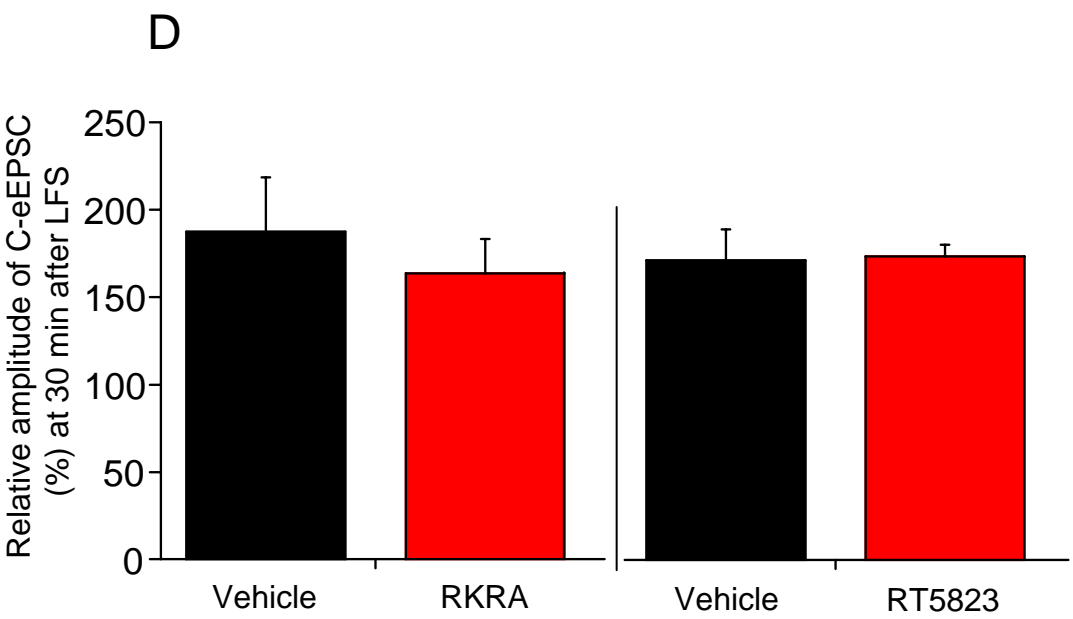

Supplement: Figure S3 — Further validation of C-fiber-evoked synaptic long-term potentiation (LTP) at spinal synapses in wild-type mice. (A) Typical examples of retrogradely labelled spinal lamina I projection neurons (lower panels) following DiI injections into the PAG (upper panel). (B) LTP was preserved upon spinal blockade of inhibitory neurotransmission with a combination of bicuculline and strychnine; n = 12 slices each. (C) Blockade of PKG-I specifically in the postsynaptic neuron via application of KT5823 in the patch pipette did not affect LTP; n = 6 slices each. (D) Quantitative summary of the magnitude of increase in c-EPSCs in the above groups at 30 min after the conditioning stimulus (LFS) over basal vales (normalized to 100). RKRARKE represents a distinct PKG-I inhibitor, which was tested in parallel experiments. (PDF) [file pbio.1001283.s003.pdf]

Supplementary Fig - 4

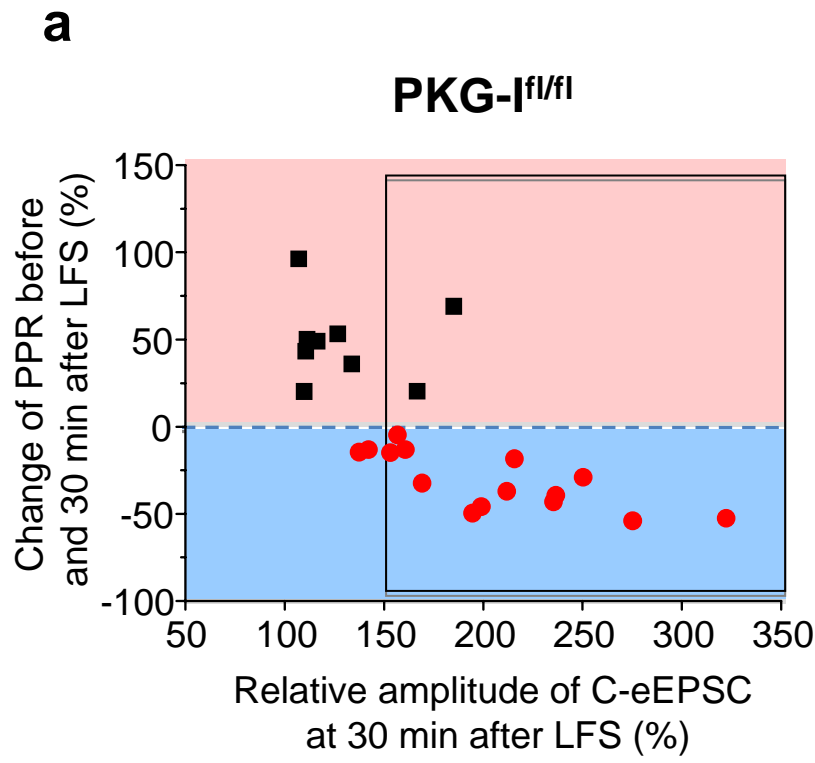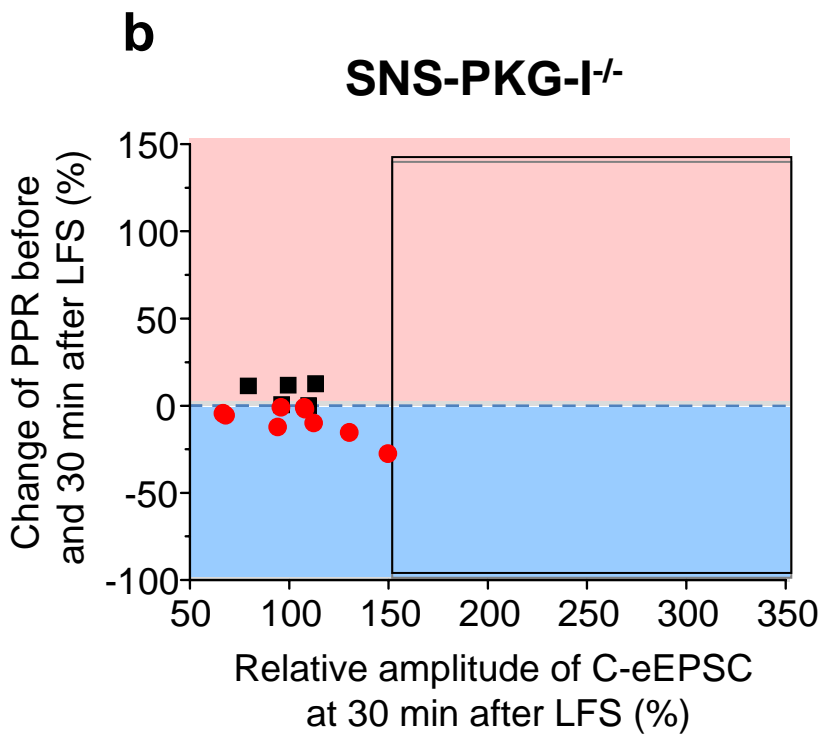

Supplement: Figure S4 — Magnitude of LTP at synapses between C-fibers and spino-PAG neurons is plotted as a function of change in PPF or PPD at the same synapse in SNS-PKG-I−/− mice and their PKG-Ifl/fl littermates. It is noteworthy that in PKG-Ifl/fl mice, synapses showing a large LTP show a decrease in PPF, indicating an increase in the release probability. An increase in PPF is only seen with a few synapses that do not show a robust LTP. These changes do not come about in SNS-PKG-I−/− mice. (PDF) [file pbio.1001283.s004.pdf]

# Supplementary Fig - 5

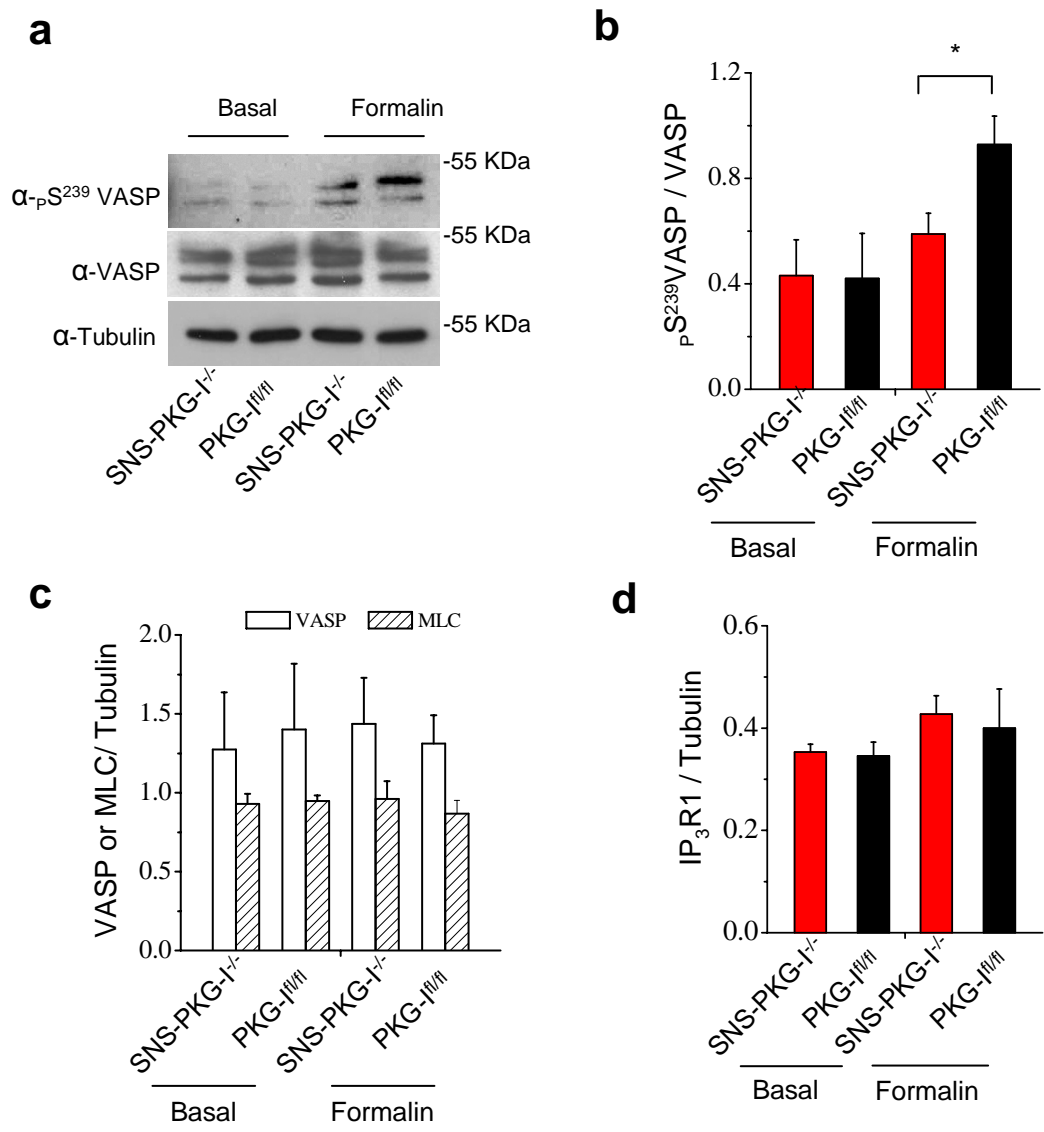

Supplement: Figure S5 — Nociceptive activity-driven phosphorylation of PKG-I substrates in DRG and spinal cord in vivo. (A, B) A typical example (A) and quantitative summary (B) of levels of VASP phosphorylated on serine 239 in L4-L5 DRGs of SNS-PKG-I−/− mice and PKG-Ifl/fl littermates in the naïve state or following formalin injection in the hindpaws. (C) Expression levels of total VASP or MLC do not vary between the above experimental groups. (D) Expression levels of total IP3R1 do not vary between the above experimental groups. In all panels, quantitative summaries are derived from three such independent experiments, each involving eight mice/group. * p<0.05, ANOVA of random measures followed by post hoc Fisher's test. Data are represented as mean ± S.E.M. (PDF) [file pbio.1001283.s005.pdf]

## Supplementary Fig - 6

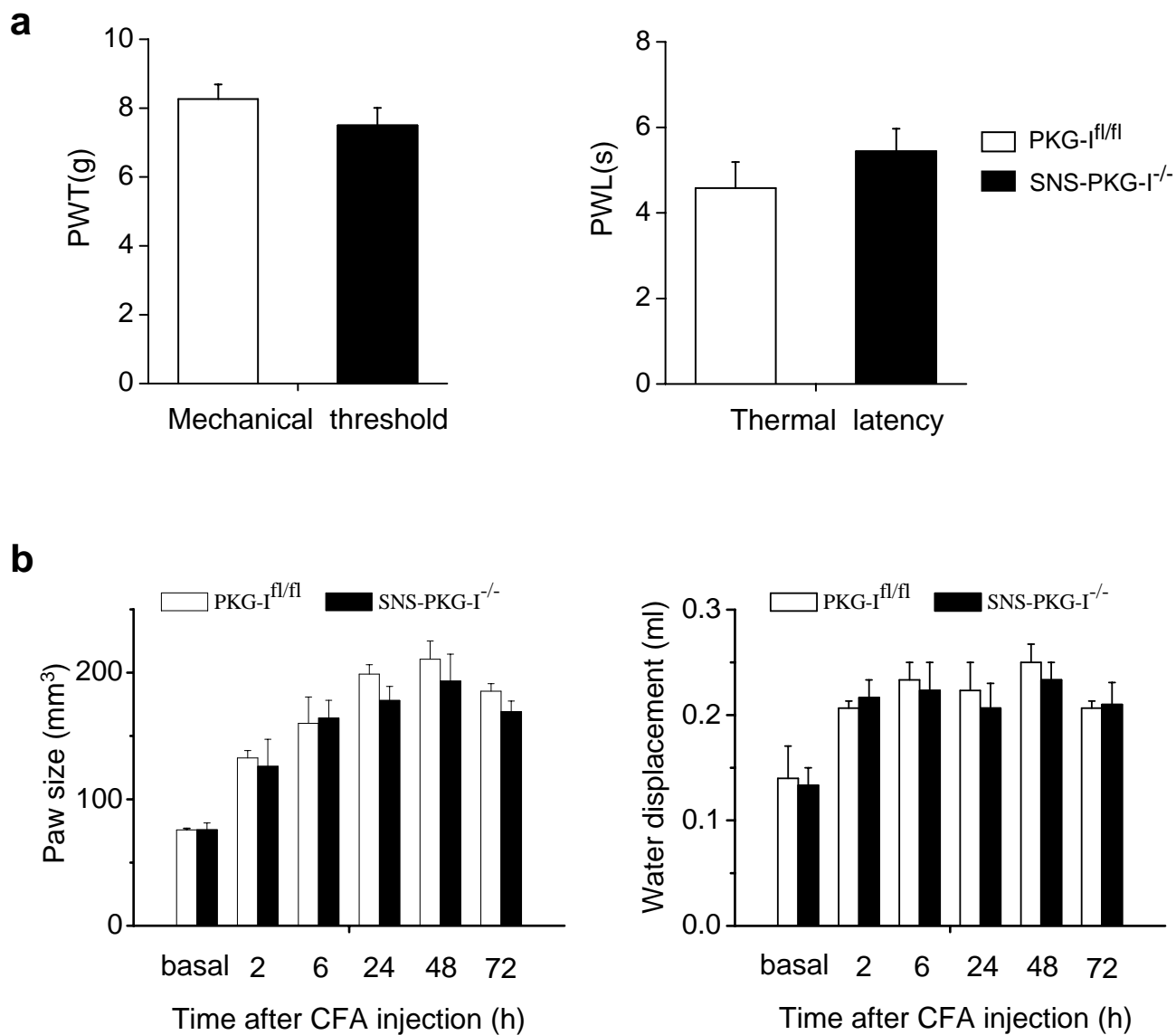

Supplement: Figure S6 — Nociceptive withdrawal responses and development of paw inflammation in SNS-PKG-I−/− mice and their PKG-Ifl/fl littermates. (A) In comparison with PKG-Ifl/fl mice (n = 8), SNS-PKG-I−/− mice (n = 8) show comparable paw withdrawal latency (PWL; p = 0.26) to radiant heat and paw withdrawal threshold (PWT; p = 0.24) to punctuate pressure. (B) Latency to fall from a rotating rod was similar in SNS-PKG-I−/− mice (n = 6) and PKG-Ifl/fl mice (n = 6). (C) The magnitude of paw edema was similar between SNS-PKG-I−/− mice and their PKG-Ifl/fl littermates following CFA-induced paw inflammation. Shown are complementary analyses of paw size in terms of paw volume (in cubic mm; length×breadth×height) and volume of water displaced by paws (in ml). All data points represent mean ± S.E.M. n = 8–10 mice per genotype or treatment group. (PDF) [file pbio.1001283.s006.pdf]
