# Supplementary material for: Presynaptically Localized Cyclic GMP-Dependent Protein Kinase 1 Is a Key Determinant of Spinal Synaptic Potentiation and Pain Hypersensitivity
Source: PLoS Biol. 2012 Mar 13;10(3):e1001283. doi: 10.1371/journal.pbio.1001283 (PMC3302842; doi:10.1371/journal.pbio.1001283)
Supplement: Figure S2 — Further evidence for normal gross development of the sensorimotor circuitry in SNS-PKG-I−/− mice. (A) Adult PKG-Ifl/fl mice and SNS-PKG-I−/− mice show similar patterns of targeting nociceptors in the spinal cord (upper panels) and the skin (lower panels) as shown by binding to TRITC-labelled Isolectin-B4 (red) and immunoreactivity for Substance P (Sub P) or CGRP (green). Level of immunoreactivity for Sub P in spinal superficial laminae was similar across genotypes (see results). (B) The density of synaptic contacts between Sub P-containing nociceptor terminals and PSD-95-expressing postsnyptic spines on spinal neurons in the spinal superficial laminae is similar across genotypes. Quantitative values on the right indicate mean percentage of association of substance P and PSD-95. (C, D) Typical examples (C) and quantitative analysis of heat pain-induced internalization of a Sub P receptor via NK1R immunoreactivity in spinal dorsal horn neurons, which was comparable across genotypes. (E) Normal spinal laminar development in adult SNS-PKG-I−/− mice and their PKG-Ifl/fl littermates. Cell bodies of spinal neurons were stained via anti-NeuN immunohistochemistry. Scale bars represent 100 µm in panel A and 2 µm in panel B. 15 µm in panel C and 100 µm in panel E. (PDF) [file pbio.1001283.s002.pdf]

Supplementary Fig. 2

a

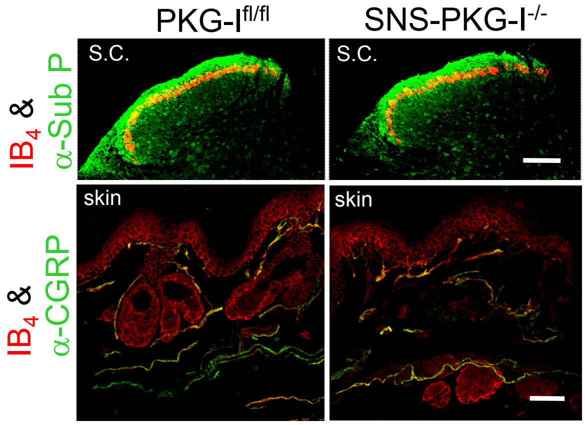

b

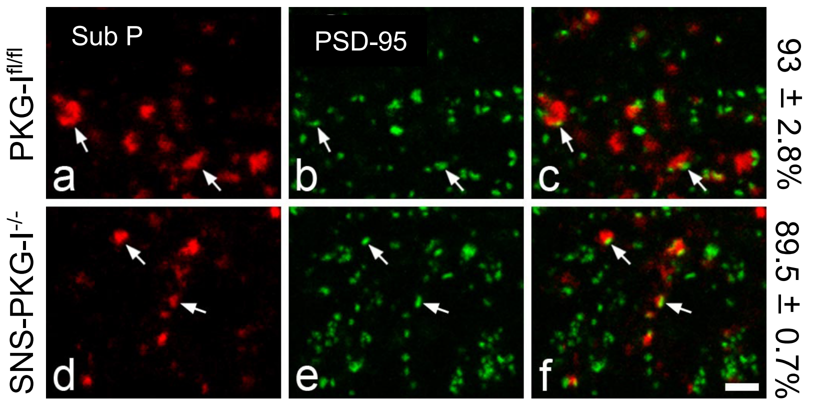

c

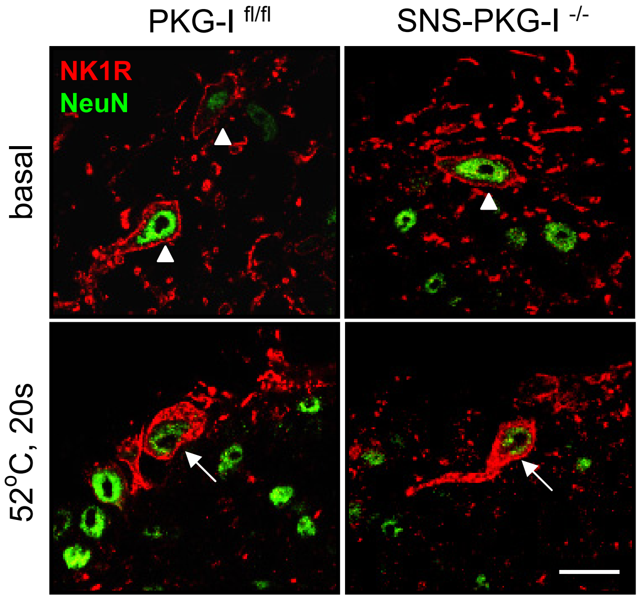

d

Genotype	Percentage of NK1 receptor internalized cells<br>number of NK1receptor internalized cells / Total number of NK1receptor positive cells X100	
	Basal	Stimulated
PKG-I $^{fl/fl}$	0.01%	73.1%
SNS-PKG-I $^{-/-}$	0.03%	77.7%

e

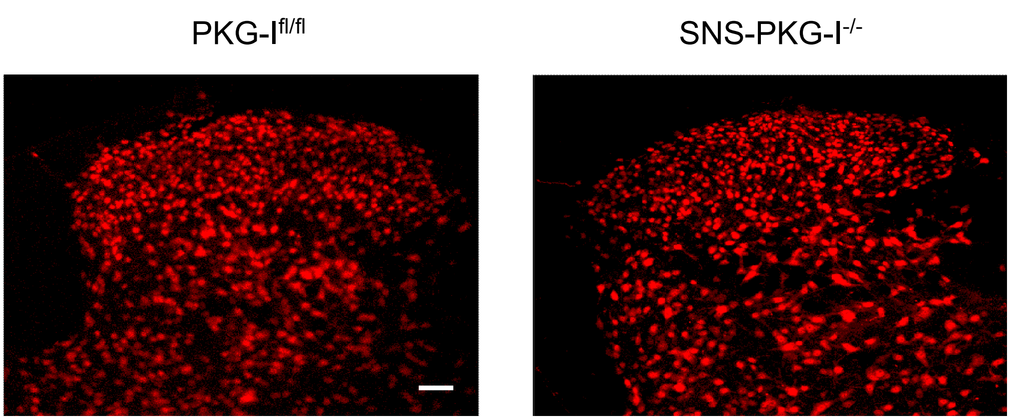

Spinal cord: anti-NeuN
