# Supplementary material for: Presynaptically Localized Cyclic GMP-Dependent Protein Kinase 1 Is a Key Determinant of Spinal Synaptic Potentiation and Pain Hypersensitivity
Source: PLoS Biol. 2012 Mar 13;10(3):e1001283. doi: 10.1371/journal.pbio.1001283 (PMC3302842; doi:10.1371/journal.pbio.1001283)
Supplement: Text S1 — Supplementary Methods and Materials. (DOCX) [file pbio.1001283.s007.docx]

**Supplementary Information, Luo et al.**

**Supplementary notes:**

1. **VASP phosphorylation**

Vasodilator-stimulated phosphoprotein (VASP) is a suitable substrate for both PKA and PKG, gets phosphorylated at ser-157, ser-239 and thr-278 (Butt et al., 1994; Smolenski et al., 1998; 2000). PKG preferentially phosphorylates at ser-239 invitro whereas invivo conditions it phosphorylates ser-239 and ser-157 with similar kinetics. On the other hand, ser-157 is preferred over ser-239 by PKA both invivo and invitro conditions. Phosphorylation of VASP at ser-157, but not at ser-239 and thr-278 leads to apparent molecular mass shift of 46kD to 50kD in SDS-PAGE and is used as marker for ser-157 phosphorylation (Smolenski et al., 1998). Probing with an antibody which specifically binds to VASP phosphorylated at ser-239 therefore gives two bands (for example, Fig. 3A upper panel), in which the upper band represents phosphorylation at both ser-157 and ser-239 and lower band represents phosphorylation only at ser-239. To determine the phosphorylation status of VASP at ser-239, we analysed the intensities of both upper and lower bands (Fig.3A upper panel) and normalised to total VASP signals (Fig. 3A middle panel).

**References:**

Butt, E., Abel, K., Krieger, M., Palm, D., Hoppe, V., Hoppe, J., and Walter, U. (1994). cAMP- and cGMP-dependent protein kinase phosphorylation sites of the focal adhesion vasodilator-stimulated phosphoprotein (VASP) in vitro and in intact human platelets. J Biol Chem 269(20), 14509–14517.

Smolenski, A., Bachmann, C., Reinhard, K., Ho¨nig-Liedl, P., Jarchau, T., Hoschuetzky, H., and Walter, U. (1998) Analysis and regulation of vasodilator-stimulated phosphoprotein serine 239 phosphorylation in vitro and in intact cells using a phosphospecific monoclonal antibody J. Biol. Chem. 273, 20029–20035

Smolenski, A., Poller, W., Walter, U., and Lohmann, S.M. (2000).Regulation of Human Endothelial Cell Focal Adhesion Sites and Migration by cGMP-dependent Protein Kinase I. J Biol Chem. 275(33), 25723-32.

**Supplementary methods:**

**DiI labelling of spinal projection neurons in vivo and patch clamp recordings:**

Mice were placed in a stereotaxic apparatus. A hole was drilled through the skull in order to insertion of a glass pipette for DiI injection. The animals received a single injection of 100 nl of 2.5% DiI into the right PAG according to coordinates derived from the atlas of Paxinos and Watson. After a 2- to 3-day survival period, transverse 350-450 μm thick spinal cord slices with dorsal roots attached were obtained. The slices were stored in an incubation solution at room temperature (in mM: NaCl, 95; KCl, 1.8; KH_2_PO_4_, 1.2; CaCl_2_, 0.5; MgSO_4_, 7; NaHCO_3_, 26; glucose, 15; sucrose, 50; oxygenated with 95% O_2_, 5% CO_2_; pH 7.4). A slice was then transferred into a recording chamber and superfused with oxygenated recording solution at 3 ml min^-1^ at room temperature. The recording solution was identical to the incubation solution except for (in mM): NaCl 127, CaCl_2_ 2.4, MgSO_4_ 1.3 and sucrose 0. All injection sites were confirmed histologically. To detect lamina I projection neurons which were labeled by DiI from the injection sites of PAG (as described above), slices were illuminated with a monochromator, and visualized with an upright fluorescence Olympus BX51WI microscope (Olympus, Japan), equipped with Dodt-infrared optics using a 40X, 0.80 NA water-immersion objective and a cooled CCD camera (TILL Photonics, Gräfelfing, Germany). Standard whole-cell patch clamp recordings were performed with glass pipettes having a resistance of 4-6 MΩ in lamina I of spinal dorsal horn. The pipette solution consisted of (in mM): K-gluconate, 135; KCl, 5; CaCl_2_, 0.5; MgCl_2_, 2; EGTA, 5; HEPES, 5 and Mg-ATP, 5, pH 7.4 with KOH, measured osmolarity 300 mOsm. QX-314 (5 mM) was added to the pipette solution to prevent discharge of APs. The electrophysiological properties of the recorded neurons were investigated in voltage-clamp modes using an EPC-10 amplifier and patchmaster (patchmaster V2.2) data acquisition software. Signals were low-pass filtered at 5 kHz, sampled at 10 kHz and analysed offline. The membrane potential was held at -70 mV. To measure excitatory postsynaptic currents (EPSCs) from labelled neurons in lamina I, dorsal root was stimulated through a suction electrode with an isolated current stimulator. Test pulses of 0.1 ms with intensity of 3 mA were given at 30 sec intervals. Aδ-fiber or C-fiber evoked EPSCs (eEPSCs) were distinguished on the basis of the conduction velocity (CV) of afferent fibers (Aδ: 2-13 m/s; C: <0.8 m/s; calculated from the latency of EPSC from a stimulus artifact and the length of dorsal root), as described previously (Nakatsuka et al., 2000; Luo et al., 2002). Aδ-fiber or C-fiber responses, respectively, were considered as monosynaptic in origin when the latency remained constant and there was no failure during stimulation at 20 Hz for 1s, or when failures did not occur during repetitive stimulation at 2 Hz for 10s (Nakatsuka et al., 2000; Luo et al., 2002). Synaptic potentiation was induced as described by Ikeda et al (2006). Low frequency stimulation (2 Hz for 2 min) was applied to dorsal root with same intensity as test stimulation. Synaptic strength was quantified by the peak amplitudes of EPSCs. The mean amplitude of 4-5 EPSCs evoked by test stimuli prior to conditioning stimulation served as a control. Significant changes from control were assessed by measuring the peak amplitudes of five consecutive EPSCs every 5 min after conditioning stimulation.

In a subset of animals, paired-pulse stimuli with an inter-stimulus interval of 110 ms (0.1 ms pulse duration, 3 mA intensity, every 30 s) were used throughout the experiment, rather than single test pulses, as described above. Paired-pulse ratio (PPR) of C-fiber evoked EPSC was expressed as the amplitude of the second C-eEPSC divided by that of the first C-eEPSC in a pair. The mean amplitude of PPR of 4-5 C-eEPSCs prior to conditioning stimulation served as a control. Significant changes from control were assessed by measuring the mean amplitude of five consecutive EPSCs at 30 min after conditioning stimulation.

**C-fiber volley recordings:** Mice were anaesthetized with a mixed solution of 1% chloresose and 17% urethane (5 ml/kg body weight, i.p.). The body temperature was maintained near 37°C using a heating blanket controlled by a rectal thermometer probe. A laminectomy was performed to expose the lumbar enlargement of spinal cord, and the dura mater was incised longitudinally. The exposed spinal cord was covered with warm paraffin oil. L4 and L5 dorsal root was dissected free for C-fiber volleys recording. Bipolar platinum hook-electrode was placed in the proximal site of the dorsal root to record C-fiber volleys in response to electrical stimulation of distal site of the dorsal root with a bipolar platinum hook-electrode (0.025-3 mA, 0.1 ms, 0.2 Hz). The distance between stimulating and recording electrodes is around 6-7 mm. C-fiber volleys response was determined on the basis of the conduction velocity and its conduction velocity slowing property following high frequency stimulation, e.g. 10 Hz, as described previously (Thalhammer et al., 1994; Gee et al., 1996; Serra et al., 1999). The amplitude of C-fiber volleys response was analyzed using Clampfit 10.0 software. Signals were filtered at 3 KHz and digitized at 33 kHz.

**Calcium imaging from cultured DRG neurons:** Mice (6-8 weeks old) were killed by inhalation of CO_2_. Bilateral spinal ganglia were removed in DMEM solution. DRG neurons were dissociated and plated on coverslips precoated with poly-lysine. At 3-5 days after culture, neurons were loaded with Fura-2 (10 μM) for 30 min. After 30 min of de-esterification period, neurons were incubated with a vital marker, Alexa 488 conjugated IB4 (10 μg/ml; Molecular probes) for live identification of small-diameter neurons for 10 min as described by Han and Simon (2006) and washed in bathing solution before Ca^2+^ imaging. This step was necessary because the DRG cultures represent a heterogeneous mix of different types of sensory neurons. Calcium signals were recorded at a rate of 2 Hz using an Axiovert135 microscope with 10X objective (Carl Zeiss Microimaging GmbH), a Sensicam CCD camera (PCO) and TILLvisION (T.I.L.L. Photonics). Agonists were applied by bath application for 10 sec and a 10 -15 min washout interval was employed between subsequent applications. F340/380 ratios were calculated as described previously.

**Immunohistochemistry:** Mice in various treatment groups were killed, perfused transcardially with 4% paraformaldehyde and spinal cord and DRGs were isolated. Vibratome sections (50 μm) of the spinal cord or Cyrostat-sections (16 μm) of the L4/L5 DRG were immunostained using standard protocol with anti-phospho-ERK1/2 antibody (Cell signaling, 1:200), anti-cre antibody (Novagen), anti-Fos antibody (Chemicon, 1:8000), anti-Isolectin B4 antibody (vector laboratories, 1:200), anti-Calcitonin gene related peptide antibody (Acric, Immunostar, 1:200), anti-Neurofilament 200 antibody (Chemicon, 1:500), anti-Substance P antibody (Chemicon, 1:200), anti-PKG-I antibody (schlossmann et al., 2000), anti-PSD-95 antibody (a gift from M. Watanabe, Hokkaido University School of Medicine, Sapporo, Japan; Fukaya et al., 2000) and anti-TrkA antibody (a kind gift from Prof. L. F. Reichardt, University of California San Francisco, San Francisco, CA, 1:2000). Immunoreactive cells in laminae I and II of the spinal dorsal horn microscopically counted in 3-4 sections per mouse from 3 mice per treatment group. Similarly, the number of immunoreactive neurons per DRG section was counted and numbers were averaged over 10 sections per mouse and 3 mice per treatment group.

**NK1 internalization in spinal cord in vivo:** Mice were anesthetized and its left hind paw was immersed in hot water at 52^0^ C for 20 seconds. After 5 min, mice were killed and perfused with 4% parafarmaldehyde. Lumbar spinal segment were isolated, sectioned and immunostained with rabbit anti-Substance P (also called NK1) receptor antibody (S8305; Sigma, 1:10,000) as described previously by Polgar et al., 2007. Only NK1-positive cells from lamina I and II were considered for analysis.

**Intrathecal delivery of drugs *in vivo*:**

To enable intrathecal delivery at the level of lumbar spinal segments in mice, a polytetrafluoroethylene catheter (PTFE Sub-Lite Wall Tubing 0.05 mm ID x 0.15 mm OD; Braintree Scientific Inc., USA) was stereotactically inserted after hemilaminectomy at S1-S2 under isoflurane anesthesia. The intrathecal catheter was attached to a silicone tube, which was externalized.

**DiI labeling of embryonic DRG afferents:**

DiI labeling of embryonic sensory afferents was done as described by Schmidt et al 2007. Mouse E13 spinal cord attached with DRGs were dissected and fixed in 4% paraformaldehyde overnight. DiI (molecular probes) dissolved in 100% ethanol was injected into DRGs using glass pipette and incubated in PBS, pH 7.4 at room temperature for 2-3 days. Whole mounts were viewed laterally using a Fluorescence microscope to visualize T-branching of DiI-labeled sensory afferents in the dorsal spinal cord.

**Pain models:**

Complete Freund’s adjuvant (CFA, Sigma Aldrich) was injected unilaterally in the intraplantar surface of the hindpaw in mice (20 µl), whereas control mice were injected with 0.9% saline, as described in details previously (Hartmann et al., 2004). Analysis of latency of paw withdrawal in response to noxious heat was done described in details (Hartmann et al., 2004) (Ugo Basile Inc.). The tail flick reflex was evoked by application of infrared heat to the underside of mouse tail and the response latency was readout from an automated device, as described previously. Mechanical sensitivity was via manual application of von Frey hairs to the plantar surface. Response frequency was calculated as the mean number of withdrawals out of 10 applications of the respective filament at 10 s intervals. The intraplantar formalin test was performed as described (Agarwal et al., 2007). Muscle pain was evoked by injecting 20μl of acidic saline, pH 4 in the gastrocnemius muscle of the flank two times at an interval of 3 days and behavioral analysis of von Frey response frequencies at the ipsilateral and contralateral paws was started at 24h until 3 weeks after the second injection (Sluka et al., 2001). Due to the large area of capsaicin-induced flare and the relatively small surface of the mouse hind paw, application of the capsaicin- evoked allodynia model in mice is challenging. We injected 10 μl of capsaicin (0.06 %) in the area between the lower flank and the ankle and observed that the flare reached up to the ankle, but not the plantar hindpaw surface. Plantar application of von Frey hairs was performed at various time points after injection of capsaicin.

**Afferent recordings in skin-nerve preparation:** A total of 32 mice (17 PKG1^fl/fl^ and 15 SNS-PKG-I^-/-^) were used in the electrophysiological recordings of nerve activity. An in vitro skin nerve preparation was used to study the properties of the afferent fibers on control conditions and in the inflamed skin 4 hours following CFA inoculation (20 μl). Animals were killed by CO2 inhalation, the saphaneous nerve was dissected with the innervated skin attached and placed in organ bath “inside-up” to expose the chorium side. The skin was placed in the oxygen-saturated modified synthetic interstitial fluid solution (123 NaCl, 3.5 KCl, 0.7 MgSO4, 1.7 NaH2PO4, 2.0 CaCl2, 9.5 sodium gluconate, 5.5 glucose, 7.5 sucrose, and 10 HEPES, in mM) at temperature of 32 ± 1°C and pH 7.4 ± 0.05, and the nerve was desheathed and teased into microfilaments to enable single-unit recording. Units were classified according to their conduction velocities, von Frey thresholds, and firing properties. Electrical stimulation of the nerve trunk was employed to calculate nerve conduction velocities and fibers were annotated as unmyelinated (C) fibers and mechanoreceptive A-δ fibers as described previously (Agarwal et al., 2007). Receptive fields were found using mechanical stimulation with a glass rod, and units were tested with ascending series of displacement mechanical stimuli ranging from 6 to 384 μm. A computer-controlled linear stepping motor (Nanomotor Kleindiek Nanotechnik) was used to apply standardized mechanical stimuli (Gangadharan et al. 2011). The raw electrophysiological data were collected with Powerlab 4.0 system and analyzed off-line with the spike histogram extension of the software.

**References for supplementary methods:**

Agarwal, N., Pacher, P., Tegeder, I., Amaya, F., Constantin, C.E., Brenner, G.J., Rubino, T., Michalski, C.W., Marsicano, G., Monory, K., Mackie, K., Marian, C., Batkai, S., Parolaro, D., Fischer, M.J., Reeh, P., Kunos, G., Kress, M., Lutz, B., Woolf, C.J., and Kuner, R. (2007). Cannabinoids mediate analgesia largely via peripheral type 1 cannabinoid receptors in nociceptors. Nat Neurosci. 10(7), 870-9.

Fukaya, M., and Watanabe, M. (2000). Improved immuno­histochemical detection of postsynaptically located PSD-95/SAP90 protein family by protease section pretreatment: a study in the adult mouse brain. J Comp Neurol. 426(4):572–586.

Gee MD, Lynn B, Cotsell B (1996) Activity-dependent slowing of conduction velocity provides a method for identifying different functional classes of C-fibre in the rat saphenous nerve. Neuroscience 73:667–675.

Han, S.K., Mancino, V., and Simon, M.I. (2006). Phospholipase Cbeta 3 mediates the scratching response activated by the histamine H1 receptor on C-fiber nociceptive neurons. Neuron. 52(4), 691-703.

Hartmann, B., Ahmadi, S., Heppenstall, P.A., Lewin G.R., Schott, C., Borchardt, T., Seeburg, P.H., Zeilhofer, H.U., Sprengel, R., and Kuner R. (2004). The AMPA receptor subunits GluR-A and GluR-B reciprocally modulate spinal synaptic plasticity and inflammatory pain. Neuron 44, 637-50.

Ikeda, H., Stark, J., Fischer, H., Wagner, M., Drdla, R., Jäger, T., and Sandkühler, J. (2006). Synaptic amplifier of inflammatory pain in the spinal dorsal horn. Science 312, 1659-62.

Luo, C., Kumamoto, E., Furue, H., Chen, J., and Yoshimura, M. (2002). Nociceptin inhibits excitatory but not inhibitory transmission to substantia gelatinosa neurons of adult rat spinal cord. Neuroscience 109, 349-58.

Nakatsuka, T., Ataka, T., Kumamoto, E., Tamaki, T., and Yoshimura, M. (2000). Alteration in synaptic inputs through C-afferent fibers to substantia gelatinosa neurons of the rat spinal dorsal horn during postnatal development. Neuroscience 99(3), 549-56.

Polgár, E., Campbell, A.D., MacIntyre, L.M., Watanabe, M., and Todd, A.J. (2007). Phosphorylation of ERK in neurokinin 1 receptor-expressing neurons in laminae III and IV of the rat spinal dorsal horn following noxious stimulation. Mol Pain, 3:4.

Schlossmann J, Ammendola A, Ashman K, Zong X, Huber A et al. (2000) Regulation of intracellular calcium by a signalling complex of IRAG, IP3 receptor and cGMP kinase Ibeta. Nature 404: 197-201

Schmidt, H., Stonkute, A., Jüttner, R., Schäffer, S., Buttgereit, J., Feil,R., Hofmann, F., and Rathjen, F.G. (2007). The receptor guanylyl cyclase Npr2 is essential for sensory axon bifurcation within the spinal cord. J Cell Biol. 179(2), 331-40.

Sluka, K.A., Kalra, A., and Moore, S.A. (2001). Unilateral intramuscular injections of acidic saline produce a bilateral, long-lasting hyperalgesia. Muscle nerve. 24(1), 37-46.

Serra J, Campero M, Ochoa J, BostockH (1999) Activity-dependent slowing of conduction differentiates functional subtypes of C fibres innervating human skin. J Physiol (Lond) 515:799–811.

Soulsby, M.D., and Wojcikiewicz, R.J. (2007). Calcium mobilization via type III inositol 1,4,5-trisphosphate receptors is not altered by PKA-mediated phosphorylation of serines 916, 934, and 1832. Cell Calcium 42(3), 261-70.

Thalhammer. J.G., Raymond. S.A., Popitz-Bergez. F.A., and Strichartz. G.R. (1994). Modality-dependent modulation of conduction by impulse activity in functionally characterized single cutaneous afferents in the rat. Somatosens Mot Res 11, 243–257.
